# Hydrophobicity-Based Force Field In Enzymes

**DOI:** 10.1021/acsomega.3c08728

**Published:** 2024-02-07

**Authors:** Irena Roterman, Leszek Konieczny, Katarzyna Stapor, Mateusz Słupina

**Affiliations:** † Department of Bioinformatics and Telemedicine, Jagiellonian University—Medical College, Medyczna 7, 30-688 Kraków, Poland; ‡ Chair of Medical Biochemistry, Jagiellonian University—Medical College, Kopernika 7, 31-034 Kraków, Poland; § Faculty of Automatic, Electronics and Computer Science, Department of Applied Informatics, Silesian University of Technology, Akademicka 16, 44-100 Gliwice, Poland; ∥ ALSTOM ZWUS Sp. z o.o, Modelarska 12, 40-142 Katowice, Poland

## Abstract

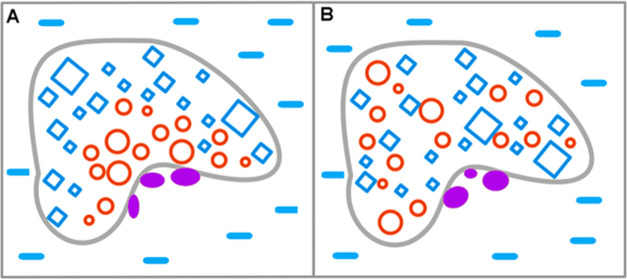

The biocatalysis process takes place with the participation of enzymes, which, depending on the reaction carried out, require, apart from the appropriate arrangement of catalytic residues, an appropriate external force field. It is generated by the protein body. The relatively small size of the part directly involved in the process itself is supported by the presence of an often complex structure of the protein body, the purpose of which is to provide an appropriate local force field, eliminating the influence of water. Very often, the large size of the enzyme is an expression of the complex form of this field. In this paper, a comparative analysis of arbitrarily selected enzymes, representatives of different enzyme classes, was carried out, focusing on the measurement of the diversity of the force field provided by a given protein. This analysis was based on the fuzzy oil drop model (FOD) and its modified version (FOD-M), which takes into account the participation of nonaqueous external factors in shaping the structure and thus the force field within the protein. The degree and type of ordering of the hydrophobicity distribution in the protein molecule is the result of the influence of the environment but also the supplier of the local environment for a given process, including the catalysis process in particular. Determining the share of a nonaqueous environment is important due to the ubiquity of polar water, whose participation in processes with high specificity requires control. It can be assumed that some enzymes in their composition have a permanently built-in part, the role of which is reduced to that of a permanent chaperone. It provides a specific external force field needed for the process. The proposed model, generalized to other types of proteins, may also provide a form of recording the environment model for the simulation of the in silico protein folding process, taking into account the impact of its differentiation.

## 1 Introduction

The issue of enzymes and their specificity is the subject of numerous papers.^1−3^ The analysis of enzyme activity focuses mainly on the construction of the active site and the forms of interaction of the substrate or competitive inhibitor.^4^ The influence of enzymes on the reaction time and its significant shortening is associated with not only local H-bonds or electrostatic interactions but also participation in the form of the presence/absence of water. Water often has to be removed from the reaction environment, which is indicated as a decisive factor for the course of the reaction.^5,6^ The analysis of the role of water is often limited to its presence/absence in the catalytic center.^7^ The key role in the catalytic process is played by the interaction of the enzyme with the substrate in its transition state.^4,8,9^ The power of binding discrimination of enzymes is being transferred to organic synthesis reactions.^10^ In addition to recognition of the functioning of already known proteins, research is moving toward the synthesis of “on-demand” enzymes. It is about the production of new enzymes that would carry out the processes we expect.^11−14^ One of the important problems in the therapy of amyloid-based diseases is the search for enzymes that digest amyloid fibrils.^15−18^

The present work focuses on the role of water as an environment in which the structure of proteins, including enzymes, is generated. The presence of water and its impact on the protein folding process directs it toward micellization, a centrally located hydrophobic nucleus with a polar shell. Such a system is common for proteins with a short polypeptide chain, including domains treated as independent structural units.^19^ Some proteins, including certain classes of enzymes, have a very complex structure with a hydrophobicity distribution different from the micelle-like. This difference may be expressed by a local deviation from the micelle-like arrangement limited to a few residues, the elimination of which reveals micelle-like structurings for the rest of the molecule. The difference may also apply to the whole protein body. This structure represents a specific force field different from that provided by water. In the case of enzymes, the force field present in the protein molecule may create favorable environmental conditions for the course of the catalysis process. Therefore, the field present in a given protein can be treated as an external force field for the reaction being carried out. The type and degree of deviation of the dispersion present in the protein in relation to the micelle-like dispersion is a specific form of recording information about the specificity of a given enzyme. The enzyme, as well as most other proteins, can be described as an “intelligent micelle.”^20^ The classic ideal micelle has only one dominant feature, limited to perfect solubility in water. On the other hand, a protein, including an enzyme in particular, apart from solubility (required for activity in an aqueous environment), has additionally encoded “knowledge” about specificity. This trace is contained in the method of dissimilarity of the hydrophobic dispersion to the micelle-like dispersion. Justification for such an interpretation of the structure determining the activity of proteins is presented in this work.

The presented model uses the term “external force field.” The expression “force field” is traditionally used to define the interactions assumed to direct the folding process toward the optimal interactions applied in programs oriented toward protein structure prediction. A force field defines the types of energy compounds expressing the nonbonding interaction (electrostatic, vdW, H-bonds, torsional potential for allowed rotations on bonds), the minimization of which leads toward the stable structure. Some force fields applied in different programs like Amber^21^ and CHARMM^22^ use different parametrization. Some programs take also into consideration so-called elastic structures (bonds stretching, bending, or even dis-planar organization of atoms in planar molecules) as components in expressing the force field in proteins.^23^

The term “external force field” is used in the model applied in this paper to express the influence of the polar water environment directing the folding process toward the micelle-like structure generation. This type of active participation of water is treated as highly important due to the water-conditioned activity of proteins and other cellular structural units. The polar water environment influences protein folding toward the generation of a hydrophobic core with a polar surface. The resultant hydrophobicity distribution in the protein body is treated as an effect of directing the activity of water. Thus, the distribution of hydrophobicity in the protein structure is treated as the effect of adaptation to conditions delivered by surroundings. It is assumed that the “internal force field,” hydrophobicity distribution in particular, is the effect of the active influence of the “external force field,” which is a water environment. Some proteins represent the hydrophobicity distribution accordant to the three-dimensional (3D) Gauss function (high concentration of hydrophobicity in the central part with low hydrophobicity on the surface) spread all over the protein body, which makes the proteins as micelle-like constructions.^19,20^ Some proteins appear to represent the distribution more or less discordant with respect to micelle-like. The degree of accordance/discordance versus the idealized 3D Gauss distribution can be assessed using the fuzzy oil drop model (FOD). The degree and form of discordance of hydrophobicity distribution versus the micelle-like assessed by FOD-M (modified) is estimated as the effect of the external force field” influence.

Large long-chain proteins appear to represent the hydrophobicity distribution highly different with respect to micelle-like organization. The membrane proteins are very good examples, the structure of which expects the exposition of hydrophobic residues on the surface to make entropic–enthalpic status optimal to stabilize the protein in the membrane environment. The membrane is treated as an external force field for structuralization of membrane proteins.

Special analysis is necessary for enzymes, where the well-defined part of this molecule plays the role of catalytic center. The usually large size of these proteins is treated as delivering special conditions for enzymatic activity. This is why the distribution of hydrophobicity as present in the protein body is treated as an “external force field” for the catalytic center. The enzyme folding process is expected to deliver the construction of a highly specific catalytic center with the appropriate local environment necessary for the catalytic reaction. The differentiation of hydrophobicity distribution in selected enzymes (representing different classes) is interpreted in the present paper as characteristics of specific “external force fields” for specific catalytic reactions. The noncompetitive inhibitors complexed in distant locations with respect to catalytic centers disable enzymes through the change of external force field arrangement. It proves the necessary presence of a protein body in enzymes to deliver the specific local environmental conditions to allow the catalytic reaction to run. The list of enzymes discussed in detail is limited, this is why Table S1 in the Supporting Information shows the large spectrum of enzymes, the characteristics of which support the interpretation given in the main body of the paper.

## 2 Materials and Methods

### 2.1 Data

Representatives of individual classes of enzymes were arbitrarily selected for analysis (Table 1).

**Table 1:** List of Enzymes Presented in the Analysis

class	class name	PDB ID	class	name
EC 1	oxidoreductases	1AEJ(24)	EC 1.11.1.5	cytochrome c peroxidase
EC 1	oxidoreductase	2C10(25)	EC 1.4.3.21	semicarbazide-sensitive amine oxidase
EC 2	transferases	1A9T(26)	EC 2.4.2.1	purine nucleoside phosphorylase
EC 2	transferases	1A47(27)	EC 2.4.1.19	cyclomaltodextrin glucanotransferase
EC 3	hydrolases	1A4M(28)	EC 3.5.4.4	adenosine deaminase
EC 4	lyases	1QCX(29)	EC 4.2.2.10	pectin lyase
EC 5	isomerases	1AG1(30)	EC 5.3.1.1.	triosephosphate isomerase
EC 6	ligases	1A82(31)	EC 6.3.3.3.	dethiobiotin synthase

These enzymes, apart from the obvious different specificity, are characterized by size variation and 3D structure, which are additional factors for comparative analysis.

The extended list of enzymes included in the analysis is available in Table S1in the Supporting Information.

### 2.2 Fuzzy Oil Drop Model

The model (FOD) used for the analysis has been described many times.^32,33^ Here, for the sake of clarity of the following interpretation, its basic assumptions will be presented.

The folding protein tends to obtain the most optimal structure in the aqueous environment, which means it tends to generate a hydrophobic core with a polar shell. The FOD model assumes that the polypeptide chain tends to form a micelle-like structure. The 3D Gaussian function was used to describe the idealized hydrophobicity distribution in such a system.
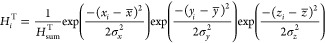
1

This function spread over the protein body (σ_*x*_, σ_*y*_, and σ_*z*_ parameters adjusted to the size and shape of the protein) represents an idealized dispersion, reflecting the one as observed in an ideal micelle. The values of this function calculated for the position of effective atoms (averaged position of atoms constituting a given amino acid) determine the level of hydrophobicity expected for a micelle-like arrangement. Its value is treated as *H*_*i*_^T^ = *T*_*i*_ later on in this paper (*T* denotes theoretical).

However, the actual dispersion is the result of hydrophobic interactions that are dependent on the intrinsic hydrophobicity of each of them and the distance between the effective atoms. To describe these interactions, the following function proposed by Levitt^34^ was used.
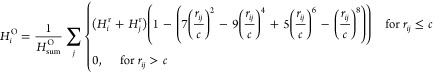
2Here, *r_ij_* is the distance between the effective atoms and c is the cutoff distance (9 Å was introduced in ref (34)). Parameter *H*_*i*_^r^ represents the intrinsical hydrophobicity of the *i*th amino acid.

Each amino acid therefore represents the actual level of *O_i_* hydrophobicity, which is a kind of collection of hydrophobicity in the close neighborhood. The value of this function is expressed as *H*_*i*_^o^ = *O*_*i*_ later in this paper (O denotes observed).

The T and O distributions (after normalization, the first factor in eqs 1 and 2) can be subjected to a comparative assessment in the form of a measure of differences between them. Here, the entropy divergence introduced by Kullback–Leibler^35^ is used.
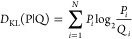
3

The level of *P_i_* examined in our model is the *O_i_* distribution, while the reference distribution (*Q_i_*) is the *T_i_* distribution. The value obtained in this way (entropy) cannot be interpreted. Therefore, a second reference distribution was proposed, R, unified, where *R_i_* = 1/*N*, where *N* is the number of amino acids in the chain.

The determined value of *D*_KL_ this time for the relation (O|R) compared with the analogous *D*_KL_ for the relation (O|T) makes it possible to determine the degree of approximation of the O dispersion to the R or T dispersions. The lower value of *D*_KL_ determines the approximation of the O dispersion to a given closer reference dispersion.

In order to eliminate the use of two parameters for the description of one object, the RD parameter was introduced.
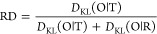
4

RD < 0.5 means the proximity of the O dispersion to the T dispersion, which is interpreted as the presence of a nucleus with a polar shell to the extent expressed by the RD value. The RD value >0.5 means the hydrophobicity distribution without the hydrophobic nucleus to the extent expressed by the RD value close to 1.0.

**Figure 1:**
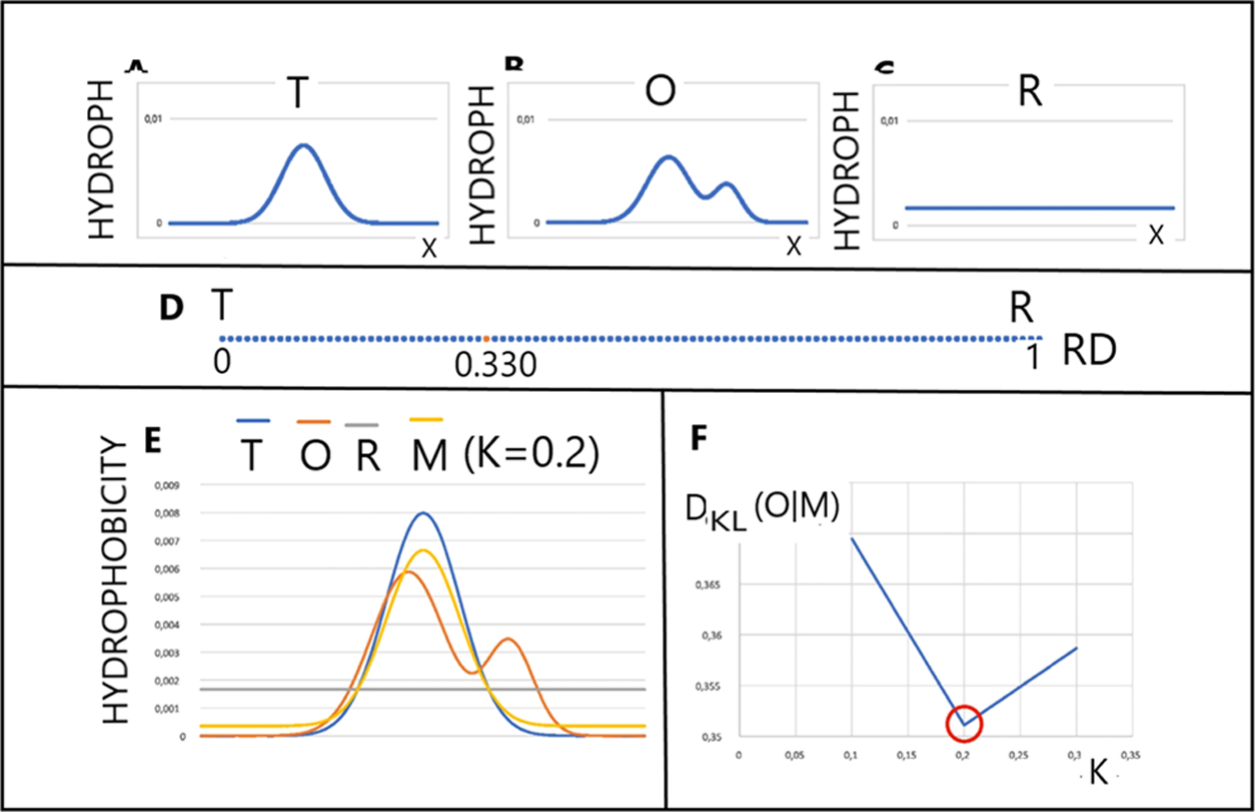
Graphic presentation of FOD and FOD-M models. (A) The idealized distribution—the theoretical one expressed by the Gauss function (eq 1). (B) The O distribution (observed one; eq 2). (C) Distribution *R* with equal distribution of hydrophobicity. (D) The RD axis with a value of 0.330 distinguished by a red dot. This value expresses the status of the *O* distribution (as shown in part (B)) with respect to reference distributions (T and R) calculated according to eq 4. (E) The *O* distribution evaluated by M at *K* = 0.2, which was found as representing the minimal *D*_KL_ for the (O|M) relation as shown in (F). (F) The minimum *D*_KL_ for the (O|M) (these values calculated according to eq 3) relation appeared to be for *K* = 0.2 (eq 6). It means the degree of modification of the aquatic environment. In this case, the modification is negligible.

A graphical visualization of the discussed interpretation is included in Figure 1. Figure 1A shows the T distribution representing the Gauss function (reduced to one dimension) treated as a reference distribution for the O distribution (Figure 1B). The second reference distribution of hydrophobicity R is shown in Figure 1C. The RD value calculated for the tree distributions appears to be equal to 0.330 (RD scale presented in the form of an axis in Figure 1D). The axis represents the range [0,1]. RD = 0 describes idealized distribution accordant to T distribution. The RD value = 1.0 represents the status of equal hydrophobicity distribution of hydrophobicity all over the object under consideration. The RD given above allows interpretation of the status as accordant with the T distribution; it means the hydrophobic core can be identified in the O distribution.

The RD value determined for any number of proteins allows for their comparative analysis from the point of view of the presence of a hydrophobic nucleus and thus, indirectly, the stability and solubility of the molecule.

The RD parameter can be determined for any defined structural unit (complex, chain, domain). A 3D Gaussian function is defined for each of these units. It is also possible to determine the role of a selected fragment of the system: chain in the complex, domain in the chain, or even a selected fragment of the chain. The RD value obtained in this way determines the participation of a given selected fragment in the construction and disruption of the structure of the hydrophobic nucleus. It turns out that in many proteins, specific fragments of the chain locally build a micelle-like system (often responsible for protein solubility), and others introduce a local disorder. This local disorder is the carrier of “information” about specificity because it turns out that the active center in the enzyme is the area with a local disorder, in particular, hydrophobicity deficiency resulting from the presence of a cavity prepared for interaction with the substrate. The kind, size, and type of disorder are precisely the record of specificity. This has been demonstrated in numerous studies.^19,36−38^ Here, such a comparative analysis was performed for selected examples of enzymes.

Calculation of RD and *K* for fragments treated as individual units requires the definition of the 3D Gauss function spread all over the defined unit. The status of the fragment as part of the structural unit is estimated using the appropriate fragments of T, O, and R. After normalization of the *T_i_*, *O_i_*, and *R_i_* values, the RD for the selected fragment can be calculated. It expresses the status of the chain fragment in a whole structural unit.

The aqueous environment is not the only one in which proteins are active. Membrane proteins, due to the different specificity of the environment (amphipathic membrane), represent the opposite tendency: the exposure of hydrophobic residues for favorable contact with the hydrophobic part of the amphipathic membrane. Membrane proteins are often channels with a central part that is almost free of packing with amino acids. Therefore, to describe this group of proteins, the function as defined in eq 5 was used in the form of a complement to the 3D Gauss function.
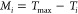
5

As studies of membrane proteins have shown so far, the proper function representing the distribution of hydrophobicity in membrane proteins is as follows (index “*n*” denotes normalization).
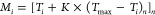
6

This formula expresses the presence of a force field coming from the water environment (*T_i_*) and the need for environment-dependent correction by the appropriate contribution of the [*T*_max_ – *T_i_*] field in the degree determined by the value of parameter *K*. Proteins such as downhill, fast-folding, ultrafast-folding show a structure described by very low RD values and *K* = 0 or a value close to 0 (*K* < 0.4).^19^ In contrast, membrane proteins show high RD values at *K* > 0.9.^−42^

Evaluation of the O distribution (Figure 1E) from the example given in Figure 1B indicates *K* = 0.2 as the modifying factor (Figure 1F). For *K* = 0.2, the *D*_KL_ value for the relation (O|M) assumes the minimum value (Figure 1F). Interpretation of such an example is as follows: the low value of RD indicates the presence of a hydrophobic core despite the visible local discrepancy. The low *K* value suggests water-only external conditions for folding (negligible low *K* value). However, the causes of local inconsistency can be further analyzed by identifying those *O_i_* positions that cause this local inconsistency. Such identification makes it possible, as it turns out in real proteins, to determine a certain purposefulness of such a system, assuming that the local discordance versus the micelle-like organization is carrying the information about the specificity.

### 2.3 Interpretation of Parameters

RD expresses the degree of adjustment of the structure of the hydrophobicity dispersion to the micelle-like system and thus is an expression of structuralization inside the protein. RD is the relative distance of O versus T with respect to reference distribution R.

*K* determines the degree of participation of the factor modifying the water environment. The higher the value of *K*, the greater the share of factors reducing the strength of the field from water, which results in the weakening of the tendency to generate a hydrophobic nucleus. At the same time, the determination of the *M* dispersion determines the type of local environment (local environment) for the reaction that takes place within a given protein and is influenced by this local external force field, which, as this work shows, is differentiated due to the catalytic reaction carried out by a given enzyme.

All of the proteins discussed in this article are characterized by these two parameters. They were determined not only for complete proteins and complexes but also for domains (if present). Characterization of the catalytic residues and the part of the protein with catalytic residues eliminated to assess their role as “information carriers” is also given. This reveals the special status of the catalytic residues and their local surroundings. The protein body with catalytic residues eliminated is treated as an “external force field” with respect to the catalytic center. The hydrophobicity distribution in the protein body representing a specific form and degree of discrepancy with respect to micelle-like is interpreted as aim-oriented to deliver appropriate conditions for the catalytic reaction.

### 2.4 Pictorial Presentation of the Applied Model

The FOD and FOD-M models are presented here in detail in graphic form to make the interpretation (especially) of the RD and *K* parameters as proposed in the FOD-M model.

**Figure 2:**
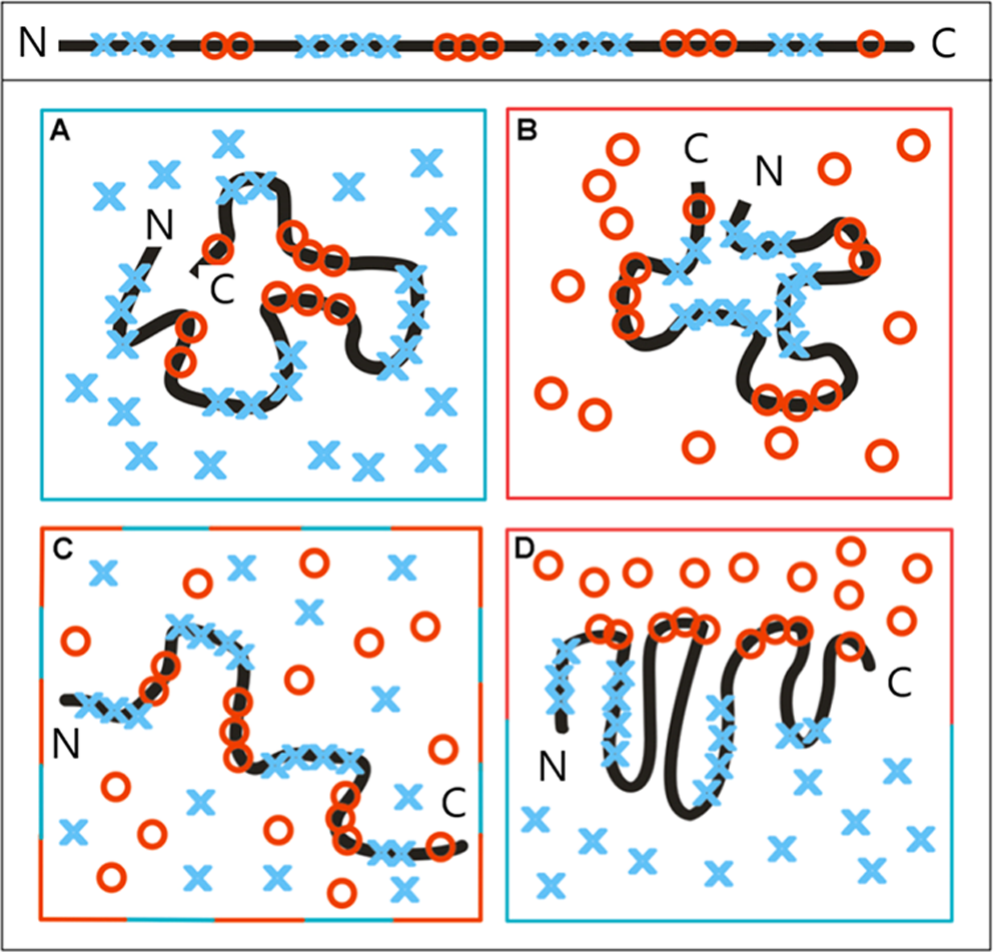
Structuralization of the polypeptide chain with hydrophobic and hydrophilic residues distributed as shown at top (A) folding in a water environment, which directs the hydrophobic residues toward the central part, with hydrophilic residues exposed on the surface hydrophobicity distribution expressed by the 3D Gauss function (low RD and low *K* values) (B) folding in a hydrophobic (nonpolar) environment, concentrating the hydrophilic residues in the central part of the hypothetical protein. Such a structure is described by high RD and high *K* values due to the absence of a hydrophobic core in the central part of the molecule. (C) An environment with a random distribution of polar (blue X) and nonpolar (red O) molecules, leading to the unfolded form of the polypeptide chain. (D) A polarized environment with hydrophobic molecules in associated form and distributed water molecules, leading to an ordered structural form, but one which is far from the 3D Gauss hydrophobicity distribution, also having high RD and *K* values. The colors in frames represent the form of the local environment.

The influence of the environment on the polypeptide chain (sequence with hydrophobic and hydrophilic residues as shown on top of Figure 2) folding as dependent on the environment is shown in Figure 2. Figure 2A illustrates the active participation of the water environment in the folding process. The polar molecules of water (blue X) direct the hydrophobic residues (red O) toward the central part to isolate them by the polar surface (blue X). The hydrophobic core is generated in such a case (Figure 2A). The 3D Gauss distribution of hydrophobicity in the protein body is reached. Thus, low *K* and low RD values describe this case.

The opposite situation is represented in Figure 2B, where the hydrophobic environment directs the folding process in the opposite (with respect to Figure 2A) direction, generating the hypothetical hydrophilic core. This case is similar to membrane proteins with high *K* and RD values.

The same amino acid sequence in a mixed environment (Figure 2C) adopts the structure that can be called unfolded due to the random distribution of polar and hydrophobic compounds. These compounds are dynamic, which makes the polypeptide chain unstable in contrast to the cases shown in Figure 2A,B. The mixture of polar and hydrophobic molecules tends to separate, as shown in Figure 2D. The highly polarized structure can be generated in such environmental conditions (Figure 2D). The parameters for this type of structure are also high as the structure represents the status without the centric hydrophobic core.

The status of the folded stable structure of the protein can be described by RD and *K* parameters. The enzymes represent the large spectrum of structural forms representing different statuses as defined by the FOD-M model. This internal force field for the enzyme molecule by itself plays the role of an external force field for the catalytic cavity and the process conducted by a particular enzyme.

**Figure 3:**
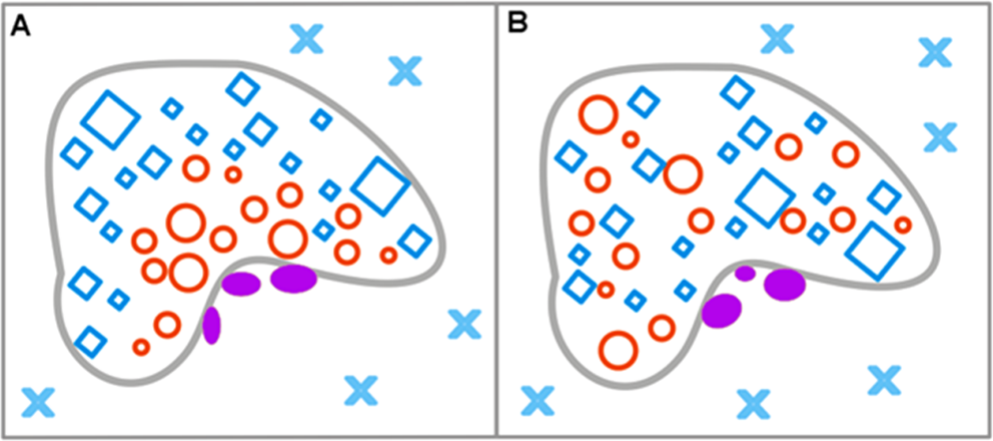
Two examples of different internal force fields that serve as external force fields for enzymatic reactions: (A) The external force field for the enzymatic reaction in the form of micelle-like distribution of hydrophobicity—low *K* and low RD. (B) The external force field for the enzymatic reaction of status expressed by high *K* and high RD values The blue squares—polar residues, and the red circles—hydrophobic residues. The gray line limits the enzyme body. The external environment—polar water (blue X) conditions. The purple ellipses—catalytic residues.

Two examples of the hypothetical status of the external force field for the enzymatic reaction are shown in Figure 3. The external force field (Figure 3A) is of micelle-like form of low Rd and *K*; the second one shown in Figure 3B represents the status of high RD and high *K* parameters. Identification of these parameters characterizes the external conditions for enzymatic reactions. The enzymes representing different classes are discussed in this paper using the methodology based on the FOD-M model to reveal the different status of the external local force field for catalytic processes.

### 2.5 Programs Used

The potential used has two possible accesses to the program.

The program allowing the calculation of RD is accessible upon request on the CodeOcean platform: https://codeocean.com/capsule/3084411/tree. Please contact the corresponding author to get access to your private program instance.

The application—implemented in collaboration with the Sano Center for Computational Medicine (https://sano.science) and running on resources contributed by ACC Cyfronet AGH (https://www.cyfronet.pl) in the framework of the PL-Grid Infrastructure (https://plgrid.pl)—provides a web wrapper for the above-mentioned computational component and is freely available at https://hphob.sano.science.

The VMD program was used to present the 3D structures.^43,44^

## 3 Results

A general description of the discussed enzymes representing different classes of enzymes is provided by a set of RD and *K* values (Table 2).

**Table 2:** List of RD and *K* Parameter Values for Complete Chains of the Discussed Enzymes and for Their Forms Devoid of Catalytic Residues^a^

	RD		*K*	
subclass	COMPL	No-CAT	COMPL	No-CAT
EC 1.11.1.5	0.475	0.467	0.4	0.4
EC 2.4.2.1	0.423	0.410	0.3	0.3
EC 2.4.1.19	0.794	0.790	1.5	1.5
EC 3.5.4.4	0.510	0.506	0.5	0.5
EC 4.2.2.10	0.707	0.701	1.0	1.0
EC 5.3.1.1	0.506	0.491	0.4	0.4
EC 6.3.3.3	0.537	0.518	0.5	0.5
EC 1.4.3.21	0.777	0.776	1.4	1.4

a COMPL-complete structure (the 3D Gauss function generated for the whole structural unit) and No-CAT-chain with catalytic residues eliminated (the RD values calculated after removing catalytic residues from the chain).

The RD values differentiate the given enzymes into three groups.

1. RD < 0.5—proteins with micelle-like structures at low *K* < 0.5
2. RD slightly above the level of 0.5 and K values compared with the group mentioned in point 1. Here, it is possible to identify residuals that introduce disorder in the sense of the FOD model. Gradual elimination of residues introducing the greatest differences between *O_i_* and *T_i_* allows for reduction of the RD value, thus revealing a part of the protein with a micelle-like structure (guaranteeing solubility) and revealing residues carrying a specificity record.
3. RD > 0.7 with high *K* values ≥1.0. Here, the deviation concerns the entire molecule, which is interpreted as a record of a specific force field.

In addition, in order to determine the role of catalytic residues, the values of RD and *K* were determined for the part of the molecule with the catalytic residues eliminated. In all examples, the RD value decreased. This means that the contribution of catalytic residues to the system is the local disorder with respect to the micelle-like form, and thus, they are carriers of information interpreted as specificity.

What is characteristic, however, is the preserved value of the *K* parameter despite the elimination of catalytic residues. This means that the influence of environmental factors applies to the whole molecule and not to shaping the specificity of the active site.

### 3.1 Proteins with Low RD and *K* Values

**Figure 4:**
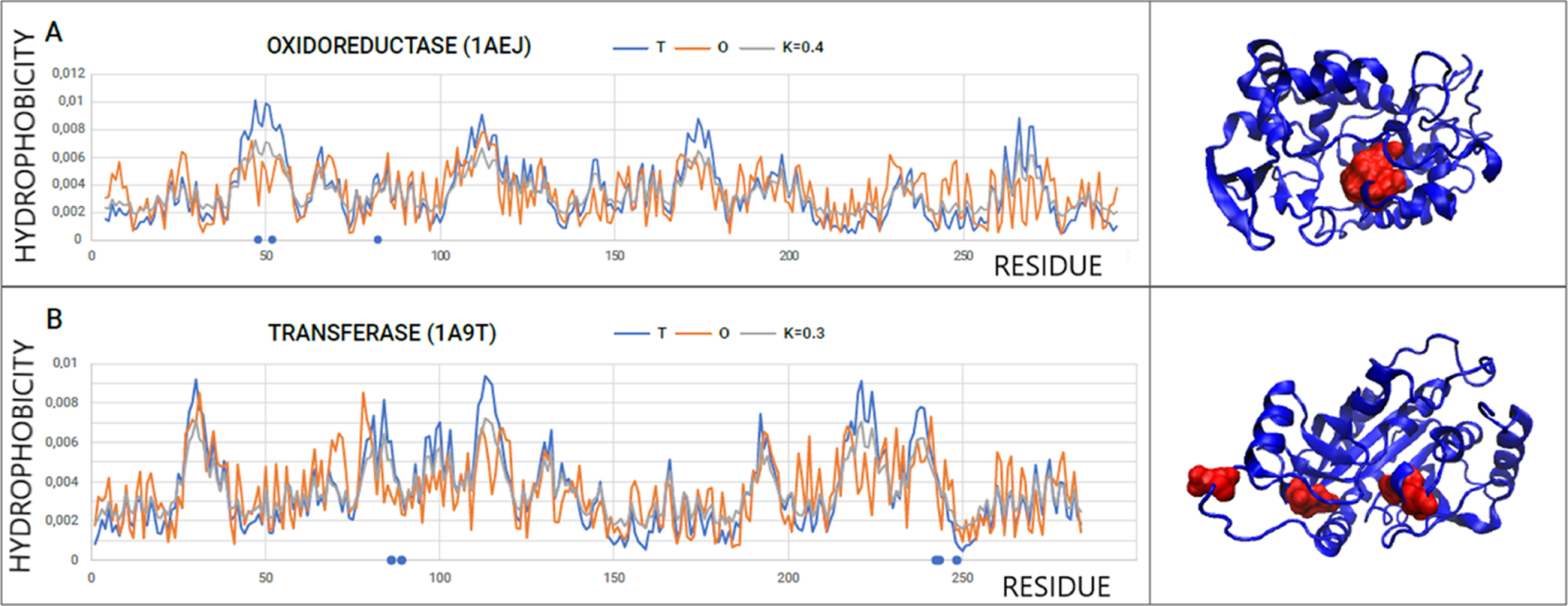
T, O, and M profiles for micelle-like enzymes together with their 3D presentation. (A) Representative of oxidoreductases and (B) representative of transferases. Blue dots on the *x*-axis represent catalytic residues shown in 3D presentation as red space-filling.

The examples of enzymes cited here exhibit a micelle-like arrangement of hydrophobicity. In the case of the oxidoreductase representative discussed here, cytochrome C peroxidase, EC 1.11.1.5 (PDB ID 1AEJ), all catalytic residues on the profiles show a local hydrophobicity deficit resulting from the presence of a cavity where the catalytic residues are located (R48, H52, and N82). The positions of the catalytic residues are characterized by a lower level of *O_i_* compared to the expected *T_i_* (Figure 4A).

Catalytic residues showing a local excess of hydrophobicity can be identified in the case of bovine purine nucleoside phosphorylase (PDB ID 1A9T), where in addition to a local deficit of hydrophobicity (residues H86, E89, T242, N243) D248 is shown by a local excess of hydrophobicity (Figure 4B).

The deficit of hydrophobicity shown by the discussed catalytic residues results from their location within the catalytic center located in the cavity.

Low values of *K* defining the distribution of M show a minimal need to modify the external force field in relation to the field generated by water (sets of T, O, and M profiles are consistent, Figure 4B). It also means that the environment generated by the protein molecule in an ordered micelle-like form creates the right conditions for the catalytic reaction. In addition, it can be speculated that the protein structure was generated with the active participation of the aquatic environment, resulting in a micelle-like arrangement.

### 3.2 Group of Enzymes with a Minimally Exceeded Threshold of RD = 0.5

The second group is representatives of isomerases (EC 5.3.1.1—triose-phosphate isomerase—PDB ID 1AG1) and ligases (EC 6.3.3.3—dethiobiotin synthase—PDB ID 1A82). These two enzymes show RD values >0.5, which means that the *O* dispersion is not adjusted to the *T* dispersion, although the difference is not large. The value of *K* only for the representative ligases is 0.5 (higher than in the previous group).

**Figure 5:**
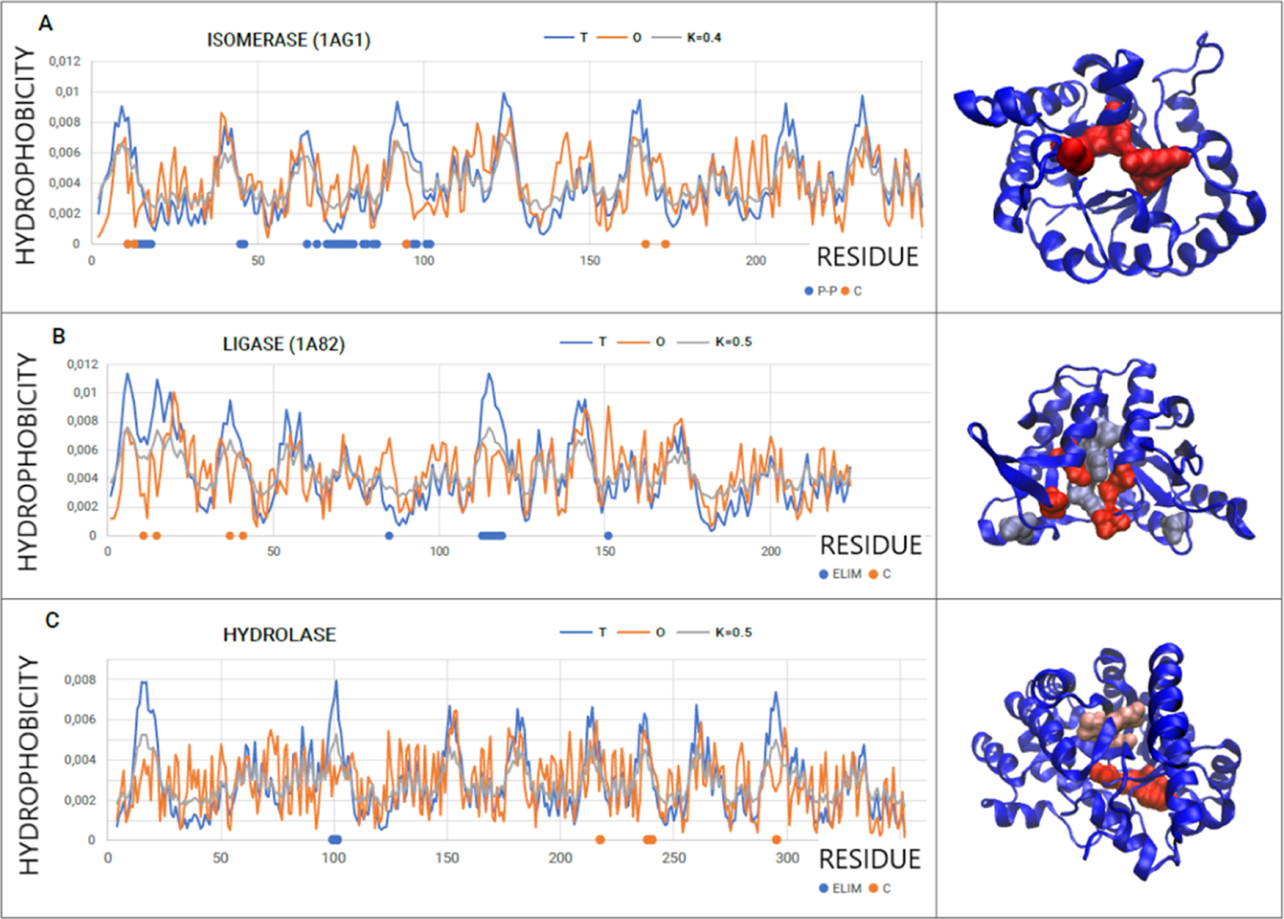
A set of T, O, and M profiles for the respective *K* values. (A) Isomerase (PDB ID 1AG1); dots on the *x*-axis: blue dots—residues involved in P–P interaction, and red dots—positions of catalytic residues. 3D representation—catalytic residues distinguished—red. (B) Ligase (PDB ID 1A82); residues eliminated to obtain RD < 0.5—blue dots (3D presentation—ice blue), and catalytic residues—red dots on the *x*-axis and red in the 3D presentation. (C) Hydrolase (PDB ID 1A4M); positions of residues eliminated to obtain RD < 0.5—distinguished as blue on the *x*-axis; in 3D presentation—pink; positions of red on the *x-*axis—catalytic residues (also red in 3D presentation).

In the case of representative isomerases, the elimination of catalytic residues (N11, K13, H95, E167, and G173) results in RD < 0.5, which means that the positions of catalytic residues have a significant impact on the local noncompliance status. In this case, the main “carriers” of information are the catalytic residues, and their location introduces local disorder (Figure 5A).

The structure of this isomerase is available in the PDB in the form of a homodimer. In addition, it is possible to analyze the status of the residues included in the interface. The positions of the residues involved in interactions with the second chain, marked on the profiles, show a local excess of hydrophobicity, which, in light of the fuzzy oil drop model, is the orientation of the folding with the encoded site ready for complexation of the second chain. The interface represents the hydrophobicity-based interaction.^45^

In the case of the ligase representative (EC 6.3.3.3—dethiobiotin synthase—PDB ID 1A82), the elimination of catalytic residues causes a decrease in the RD value (which proves the specific position within the molecule), although the RD does not decrease below 0.5 (Figure 5B). This means that other residues introduce additional local maladjustment (local disorder in relation to micelle-like). Figure 5B shows additionally eliminated residues (red dots), the elimination of which results in a value of RD = 0.488. It can be assumed that these residues also constitute a specific record of the specificity of the protein in question. The analysis of the 3D structure suggests the participation of residues additionally removed as components of the cavity located in the very center of the molecule and the residue 151C on the surface showing excess hydrophobicity (on the 3D presentation, the leftmost residue is highlighted in ice blue).

A representative of the hydrolases and in particular adenosine deaminase (EC 3.5.4.4—adenosine deaminase—PDB ID 1A4M) also shows a minimal RD threshold (RD = 0.510). Elimination of catalytic residues (217E, 238H, 240Y, and 295D) reduces this value to 0.506. Elimination of the E99, V100, and R101 residues (obvious deficit of hydrophobicity) results in obtaining the value of RD = 0.496. It should be noted that the identification of such additional residues suggests their participation in the information carried and thus their participation in specificity (Figure 5C).

### 3.3 Examples of Enzymes with High RD and *K* Values

Another example of an enzyme is cyclomaltodextrin glucanotransferase representing the class of hydrolases and, in particular, the subclass EC 2.4.1.19.

**Figure 6:**
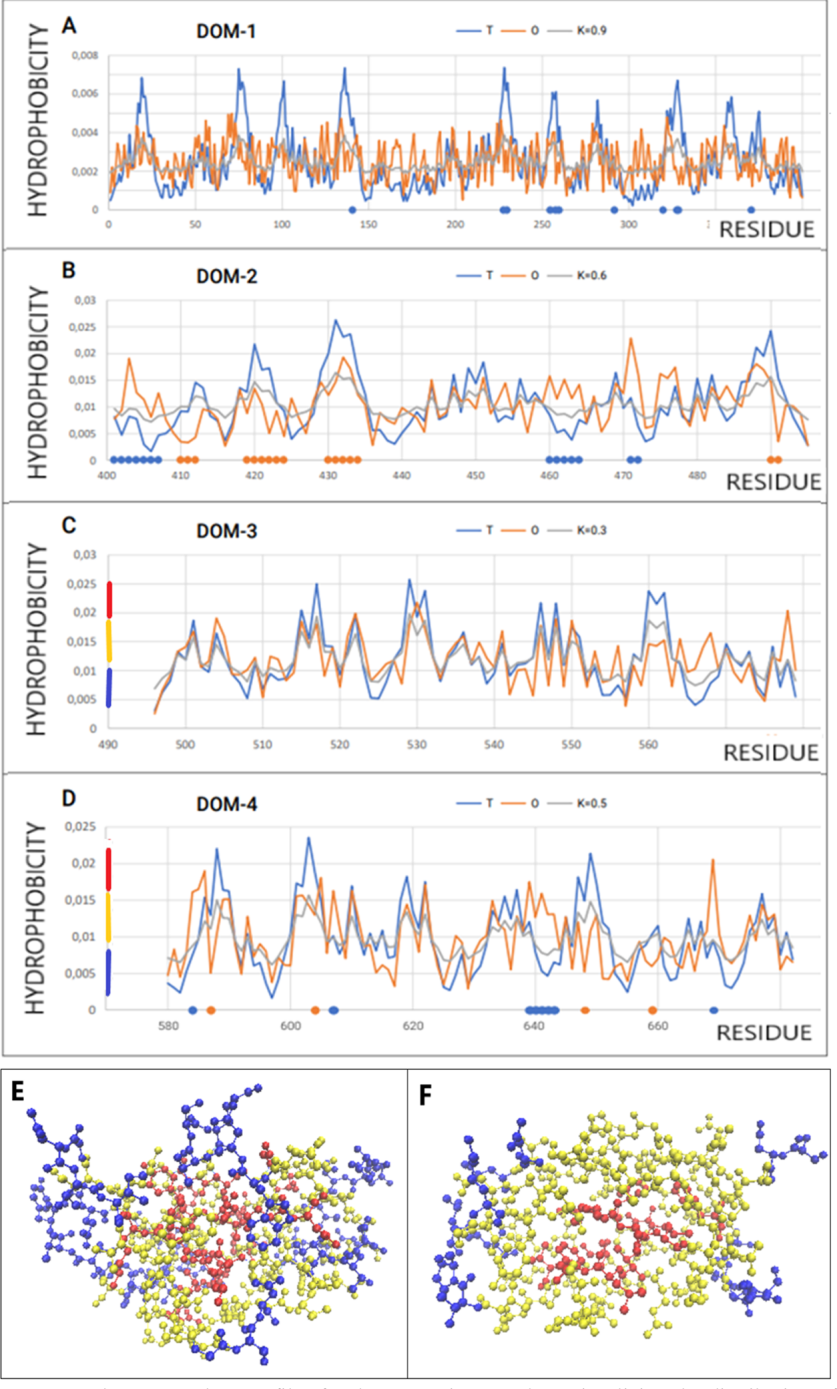
T, O, and M profiles for the respective *K* values visualizing the distribution of hydrophobicity in the transferase (PDB ID 1A47) domains. (A) Dom1: blue dots on *X*-axis—catalytic residues (B) Dom2: blue dots—residues of hydrophobic excess and red dots—residues with hydrophobicity deficiency. (C) Dom3: high accordance of all compared profiles T, O, and M—*K* value very low 0.3. The vertical lines represent the ranges of hydrophobicity distinguished in the 3D presentation in (E). (D) Dom4: residues with local hydrophobicity excess—blue dots, and residues with local hydrophobicity deficiency—red dots. The vertical lines represent the ranges of hydrophobicity distinguished in the 3D presentation in (F). (E) 3D presentation of Dom3 with levels of hydrophobicity differentiated according to ranges distinguished in (C). (F) 3D presentation of Dom4 with levels of hydrophobicity differentiated according to ranges distinguished in (D).

This protein is composed of a single chain (683 aa) in which CATH identifies four domains (called Dom#). The status of the entire chain and the domains present in it is given in Table 3 and Figure 6. The characteristic feature is the presence of all catalytic residues (11:141H, 228R, 230D, 255T, 258E, 260F, 292Q, 320D, 328H, 329D,371D) within Dom1 (Figure 6A).

**Table 3:** Parameters Characterize the Status of a Complete Molecule and its Domains^a^

	part of chain^b^		individual unit^c^	
transferase (PDB ID 1A47)	RD	*K*	RD	*K*
complete chain			0.794	1.5
Dom1 (1–400)	0.695/0.686	0.8/0.8	0.672/0.652	0.9/0.8
Dom2 (401–495)	0.795	1.7	0.607	0.6
Dom3 (496–579)	0.842	1.6	0.461	0.3
Dom4 (580–682)	0.671	0.8	0.535	0.5

a Double values for Dom1 express the second position status after elimination of catalytic residues.

b Part of chain— the column presents the RD and *K* parameters treating the chain fragments as part of the complete chain.

c Individual unit—the chain fragments (as given in the right column) for which the appropriate 3D Gauss function was generated.

The complete chain status turned out to represent a hydrophobicity distribution far from micelle-like (RD > 0.7 at *K* = 1.6). This means that a hydrophobic core common to the entire structure cannot be identified in the structure. In addition, it can be stated that the structure represented by this protein is not the effect of the aquatic environment but of a significantly modified environment.

Domains treated as individual structural units (3D Gaussian function determined for each domain) show different statuses. Dom3 turns out to represent a micelle-like arrangement (Figure 6C). It can be assumed that this domain obtained its structuring under the influence of the aqueous environment, which led to the formation of a hydrophobic nucleus and a polar shell within this particular domain. Thus, it can be assumed that this domain structured itself according to the FOD model. The remaining domains show far from micelle-like status. The highest degree of maladjustment in this system is shown by Dom1, where the active center is present. This domain cannot be a product of the aqueous environment but rather of a significantly modified environment at *K* = 1.6. The other two domains represent an intermediate status with RD values >0.5 and relatively low *K* values (relative to the whole structure).

The assessment of the status of domains as components of the entire structure is very diverse. Dom3, which as an independent structural unit shows a micelle-like order in the context of the whole structure, turns out to be the most unsuitable for the whole-chain arrangement. It is somewhat obvious because the component, which has its own hydrophobic nucleus at the peripheral position in its structure, does not adapt to the arrangement of the entire chain (Table 3).

The comparable status of Dom1 is characteristic of the interpretation of this domain as an independent structural unit and as part of a complete system. On the other hand, Dom2 and Dom3 bring with their status the highest degree of mismatch of the hydrophobicity dispersion in relation to the dispersion induced by the presence of an external force field originating from water.

Determining the RD value for a chain devoid of catalytic residues is intended to identify the status of these residues in relation to the entire structure. The elimination of catalytic residues, which in the vast majority represent a status inconsistent with the expected, is due to the fact of their location in the cavity, but it can also be treated as a factor carrying information for a given system.

In the case of the enzyme discussed here, the RD values after elimination of the catalytic residues reduce the RD value but to a small extent. This means that these residues fit into the status of their local environment (cavity) and the status of the entire molecule. Comparable *K* values for structures devoid of catalytic residues suggest the participation of the environment in the construction of the whole system and not individual residues, including catalytic residues in particular.

Dom1 appears to represent the status of the highest RD and *K* (Figure 6A) in comparison to other domains in this enzyme. Dom2 (Figure 6B) appears comparable to Dom4 (Figure 6D) with respect to RD and *K* values.

Analysis of T, O, and M profiles for Dom3 (Figure 6C) is a typical example of a domain (according to the definition, a domain is a part that folds independently of the other parts of the chain), whose structuring was carried out in accordance with the influence of the aqueous environment. There are locations with a local excess and deficit of hydrophobicity. The RD value for this domain, however, indicates a global order showing the presence of a hydrophobic nucleus and a polar surface shell.

Two domains (Dom3 and Dom4) appear to represent the O distribution accordant with the T distribution at a relatively high level. This is why these two domains can be used to visualize the distribution of hydrophobicity in 3D presentation (Figure 6E,F).

**Figure 7:**
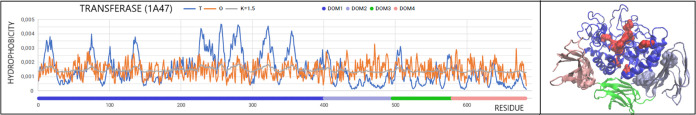
Characteristics of the transferase (PDB ID 1A47): T, O, and M profiles—domains marked on the *x-*axis together with a 3D presentation with domains distinguished in colors as distinguished on the *x*-axis. Dom1: navy blue with red residues—catalytic residues. Dom2: ice blue—space-filling selected—excessive hydrophobicity. Dom3: green—RD < 0.5. Dom4: pink—space-filling—excessive hydrophobicity.

Dom4 shows an RD value >0.5, although the analysis of the T, O, and M profiles (Figure 6D) indicates one location with a significantly increased level of hydrophobicity. Eliminating this segment results in RD < 0.5. The role of this segment (Figure 7) presented in the 3D presentation is responsible for the interaction with Dom3.

The analysis of the 3D structure of this enzyme reveals the aim orientation in the structure of this protein. Dom1 is entirely responsible for the catalytic action (all catalytic residues located within this domain). The high RD value for this domain suggests a significant departure from the micelle-like system. Such a state in an aqueous environment may turn out to be unstable. The high value of *K* (comparable to the membrane protein—rhodopsin^40^) suggests a significant modification of the environment for this domain. An external membrane force field is needed to maintain the high *K* dispersion in the membrane. Here, in the discussed example (protein works in an aqueous environment), a source of an external force field is needed. In this particular example, three domains perform these roles. The complete protein body with the present domains acts as a provider of an external force field, stabilizing the structure not generated by the aquatic environment. The entire protein body, including the three domains present, can be treated as a permanent chaperone that stabilizes Dom3 structuring while providing external conditions that are conducive to the catalysis process.

It can be speculated that Dom3 is an element of stabilization in response to the aqueous environment. All three domains play the role of a factor modifying the external force field, stabilizing the structuralization essential for the catalytic properties of Dom1. Domains 2–4 can be described as a permanent chaperone that watches over the stabilization of the protein, including the stabilization of Dom1 in particular.

### 3.4 Lyase Representative

Another example of an enzyme characterized by high RD and *K* values is the lyase representative of E.C.4. and, in particular, the subclass EC 4.2.2.10, which is pectin lyase (PDB ID 1QCX).

**Figure 8:**
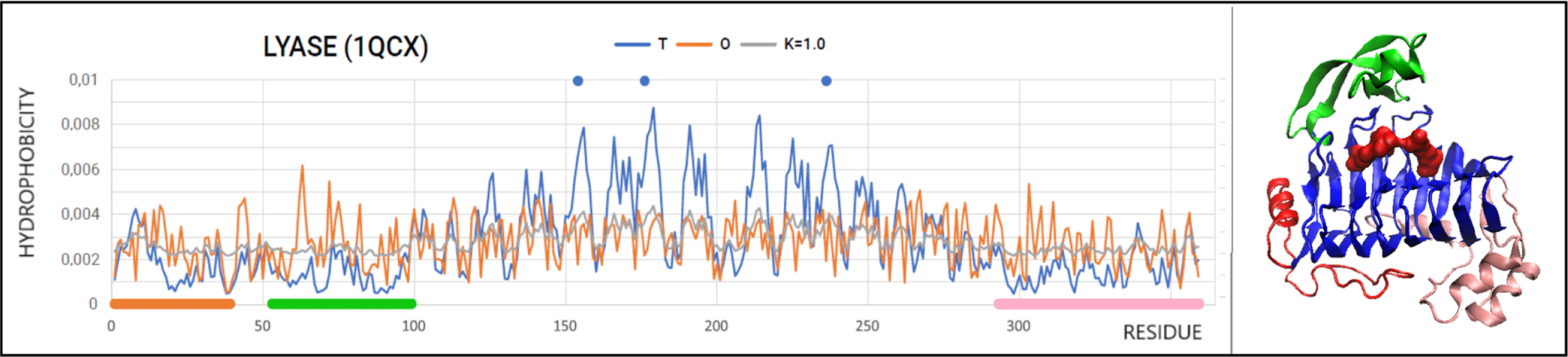
T, O, and M (*K* = 1.0) profiles showing the status of pectin lyase (PDB ID 1QCX) with highlighted fragments as shown in the 3D presentation. Points on the upper line—positions of catalytic residues. The lines on the *x*-axis—pseudodomains colored according to 3D presentation. The pseudodomains are defined as follows: (1–39) red—random coil section with a fragment of the α-helix, (53–99) green—section not included in the solenoid, and (293–359) pink—C-terminal fragment in disordered form with two helical segments. Residues distinguished as red space-filling—catalytic residues (blue dots on the top line above the T, O, and M profiles). The status of the enzyme in question is described by high values of RD = 0.7 and *K* = 1.0, which means that the structure of this protein is not based on the micelle-like model.

This enzyme is characterized by the presence of a supersecondary structure in the form of a solenoid. The CATH classification does not identify domains, but for the purposes of the present work, it has been proposed to distinguish the following parts (pseudodmains): N-terminal (Figure 8—red fragment), central not included in the solenoid (Figure 8 —green fragment) and C-terminal (Figure 8—pink fragment) and the main stem in the form of a solenoid (Figure 8—blue fragment).

**Figure 9:**
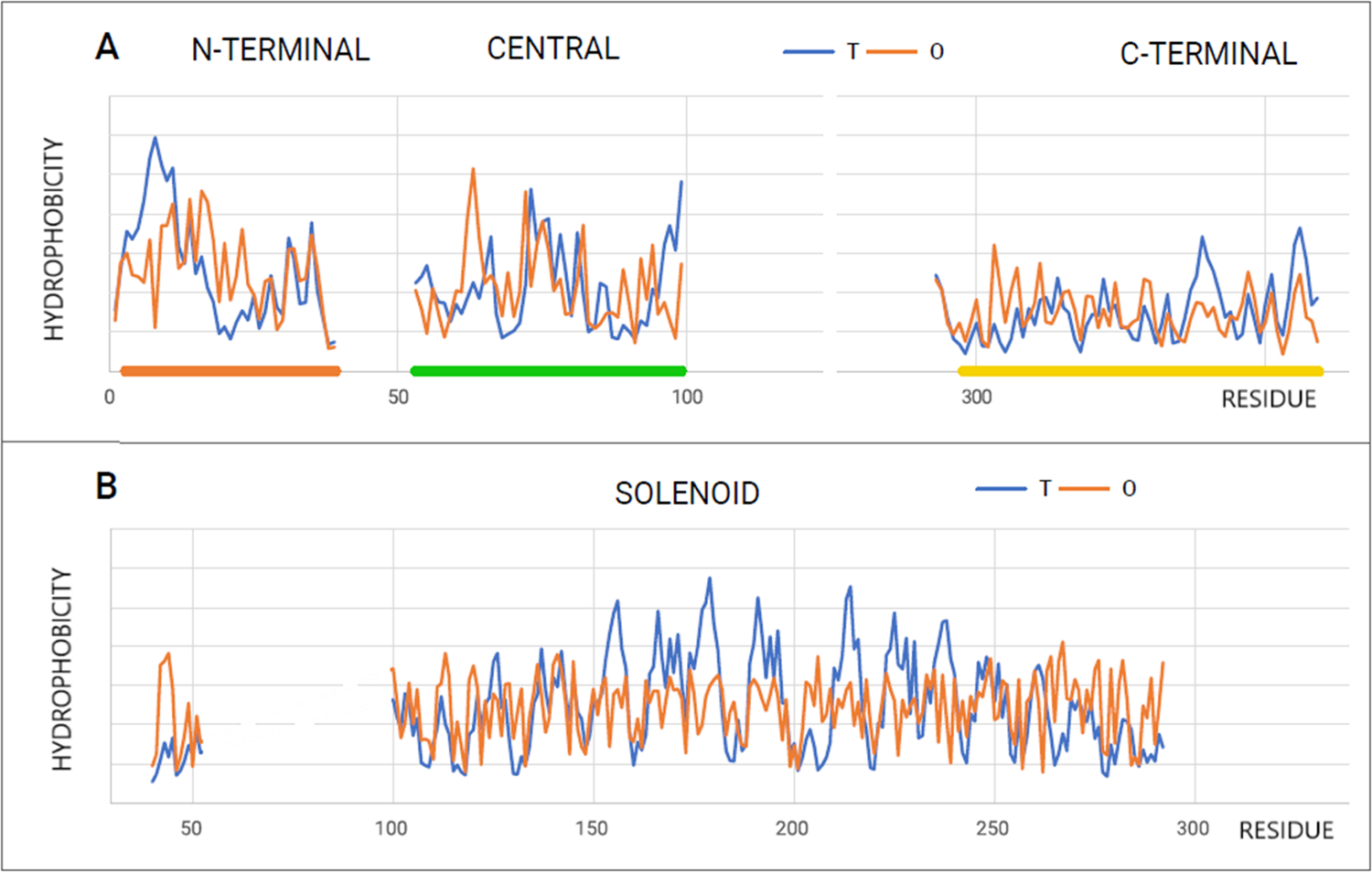
Set of T, O, and M lyase profiles for the chain sections highlighted in Figure 8. The highlighted sections are treated as construction components within the framework of the discussed protein. Coloring as in Figure 8. (A) Detailed presentation of chain fragments (solenoid excluded)—the lines on the horizontal axis distinguish the chain fragments according to colors used in Figure 8B. (B) Detailed presentation of T, O, and M profiles for the solenoid part.

The analysis of the set of T, O, and M profiles reveals the type of discrepancy between the O distribution and the *T* distribution in the distinguished sections treated as independent structural units (Figure 9). The N- (red) and C-terminal (orange) sections and the (53–99—green) fragment show a significant excess of hydrophobicity. The part in the form of a solenoid shows a significant deficit of hydrophobicity in relation to its expected concentration in the central part of the molecule.

An M dispersion that is significantly close to a straight line (oscillating around a straight line) reveals the presence of a micelle-like order. This means generating a local force field with cyclically changing characteristics (level of hydrophobicity), thus creating an environment for the catalytic reaction.

Here, as in the case of cyclomaltodextrin glucanotransferase representing the class of hydrolases, and in particular the subclass EC 2.4.1.19 (PDB ID 1A47), there is no clearly defined local incompatibility, the elimination of which would separate a part of the molecule with a micelle-like structure. On the one hand, the molecule can be characterized as significantly failing to meet the ordering conditions with a centrally located hydrophobic nucleus. However, this type of decomposition can also be interpreted as an external force field suitable for the catalytic reaction to be carried out.

It should be noted that all three catalytic residues are located within the solenoid, i.e., the part showing the highest degree of deviation from the hydrophobicity dispersion toward the micelle-like. The solenoid is therefore a significant structure providing a specific force field against the catalytic residues.

Status of the highlighted sections: N-terminal RD = 0.569, Central RD = 0.598; C-terminal RD = 0.604, Solenoid RD = 0.672 clearly expresses the need for a field significantly different from that which would be generated by the aqueous environment. On the other hand, this type of field turns out to be appropriate for this particular enzyme and the reaction it carries out.

It is noteworthy that the N-terminal fragment with the lowest RD value contains an α-helical segment in its structure. It plays a significant role in terminating the propagation of the solenoid, which (similar to the amyloid fibril) can theoretically propagate to an unlimited extent. The helical section shows an RD of 0.253, which proves that the hydrophobicity is ordered in accordance with the expectations of the aqueous environment. This helical section is typically amphipathic in nature, where one side of the helix exhibits enthalpically favorable properties toward water (polar side), while the side facing the solenoid exhibits hydrophobic characteristics. This form of a short helix has been proposed as a means of terminating the propagation of amyloid fibrils.^46^

### 3.5 Primary Amine Oxidase (PDB ID 2C10)

Another example discussed in this work, with high values of RD and *K* parameters, is human copper-containing amine oxidase EC 1.4.3.21—primary amine oxidase (PDB ID 2C10).^25^ In addition to the Cu^2+^ ion present in the catalytic center, a unique organic cofactor, 2,4,5-trihydroxyphenylalanine quinone (TPQ), is also necessary for biological activity.

The reaction catalyzed by the enzyme in question is as follows.
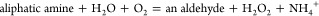
7

In the structure of this enzyme, the CATH classification identifies three domains. The active center and the complexation site of the Cu^2+^ ion are located in Dom3.

This enzyme is considered an example of a protein exhibiting enzymatic activity.^47^

Therefore, the analysis of its field may be useful for the use of its properties to design other de novo enzymes.^25,48,49^ The characteristics of the discussed enzyme based on the FOD-M model are shown in Table 4.

**Table 4:** Set of Parameters Describing the Status of the Dimer and the Individual Chains and Domains^a^

	individual chains/in complex		catalytic residues eliminated		RD	
primary amine oxidase	RD	*K*	RD	*K*	P–P	no P–P
chains A + B	0.777	1.4	0.776	1.4	0.762	0.766
chain A	0.778/0.779	1.1/1.4	0.776	1.1	0.828	0.765
chain B	0.777/0.774	1.1/1.4	0.776	1.1	0.892	0.765
DOM1 (41–165)	0.599/0.691	0.5/1.4				
DOM2 (166–297)	0.541/0.669	0.4/1.0			0.760	0.422
DOM3 (322–728)	0.723/0.636	0.8/0.7	0.723	0.8	0.813	0.684
A-DOM3 + B-DOM3	0.744/0.633	1.4/0.7	0.744	1.4	0.802	0.716

a The characteristics of two juxtaposed Doms3 are also given. Amino acids whose role is discussed as critical for biological activity (469L or 323N) are located in Dom3.^47^

**Figure 10:**
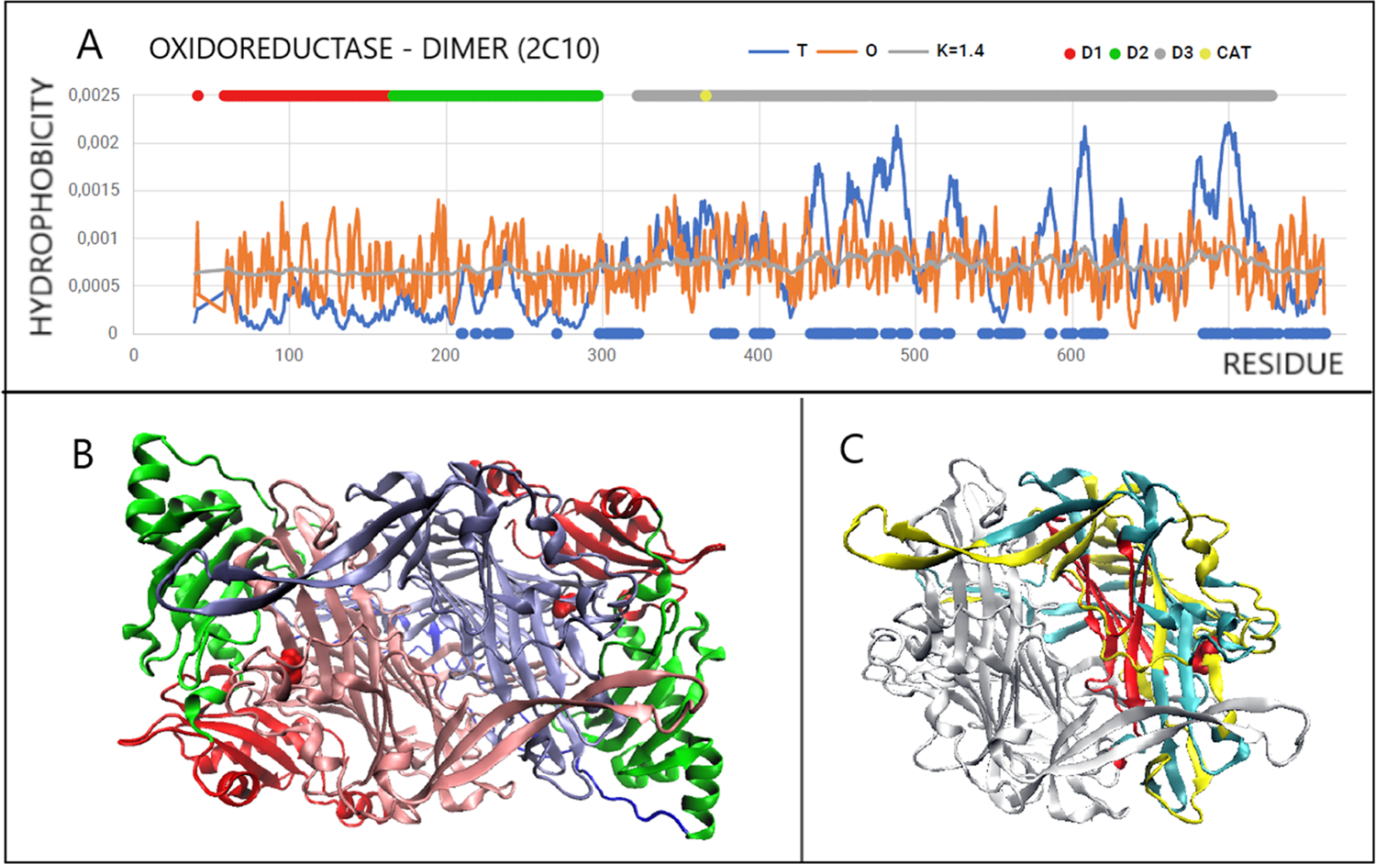
Characteristics of oxidoreductase (PDB ID 2C10). (A) T, O, and M profiles for *K* = 1.4. Blue dots on the *x*-axis: residues engaged in the P–P interaction. The top line—domains distinguishing. Yellow dot—catalytic residue. (B) 3D presentation of the dimer with domains distinguished (colors according to (A)). (C) 3D presentation of the dimer: chain A—cyan with fragments distinguished: red—hydrophobicity deficiency (as shown in A) and hydrophobicity excess—yellow (as shown in A). Catalytic residue (386D)—red space-filling. Chain B—white.

The juxtaposed profiles of T, O, and M representing two chains included in the homodimer indicate a high symmetry of the system. All profiles coincide, which means that exactly the same distribution in both chains (Figure 10A) shows superimposed profiles originating in two chains. A high RD value and a very high *K* value indicate structuralization requiring significant participation of nonaqueous factors in shaping the structure of this enzyme. It also means a highly specific local field provided by this protein, constituting an environment for the catalytic reaction.

The 3D presentation revealing different statuses from the point of view of local redundancy and local deficit (Figure 10B,C) reveals the specificity and complexity of the field in the immediate vicinity, probably also introducing the possibility of local structural changes.

### 3.6 Role of Disulfides

The hydrophobic nucleus and disulfide bonds are responsible for stabilization of the tertiary structure. In the group of proteins discussed, there are two, where domains have been identified and two in which disulfide bonds are present. These proteins are characterized by a long chain length. There is also one protein in which domains are distinguished and where disulfide bonds are present.

The status of the chain segments expressed by the parameters taken into account by the FOD model is listed in Table 5.

**Table 5:** Values of RD and *K* Parameters Determined for Sections Defined by the Positions of Disulfide Bonds^a^

fragment	RD	*F*
oxidoreductase (706 aa)	0.778	1.1
41–748	0.779	1.1
198–199	0.495	0.2
404–430—Dom3		
734–741	0.718	1.0
lyase (359 aa)	0.707	1.0
63–82	0.633	0.6
72–206	0.700	0.8
303–311	0.551	0.3

a These bonds are present in oxidoreductase (PDB ID 2C10) and lyase (PDB ID 1QCX).

**Figure 11:**
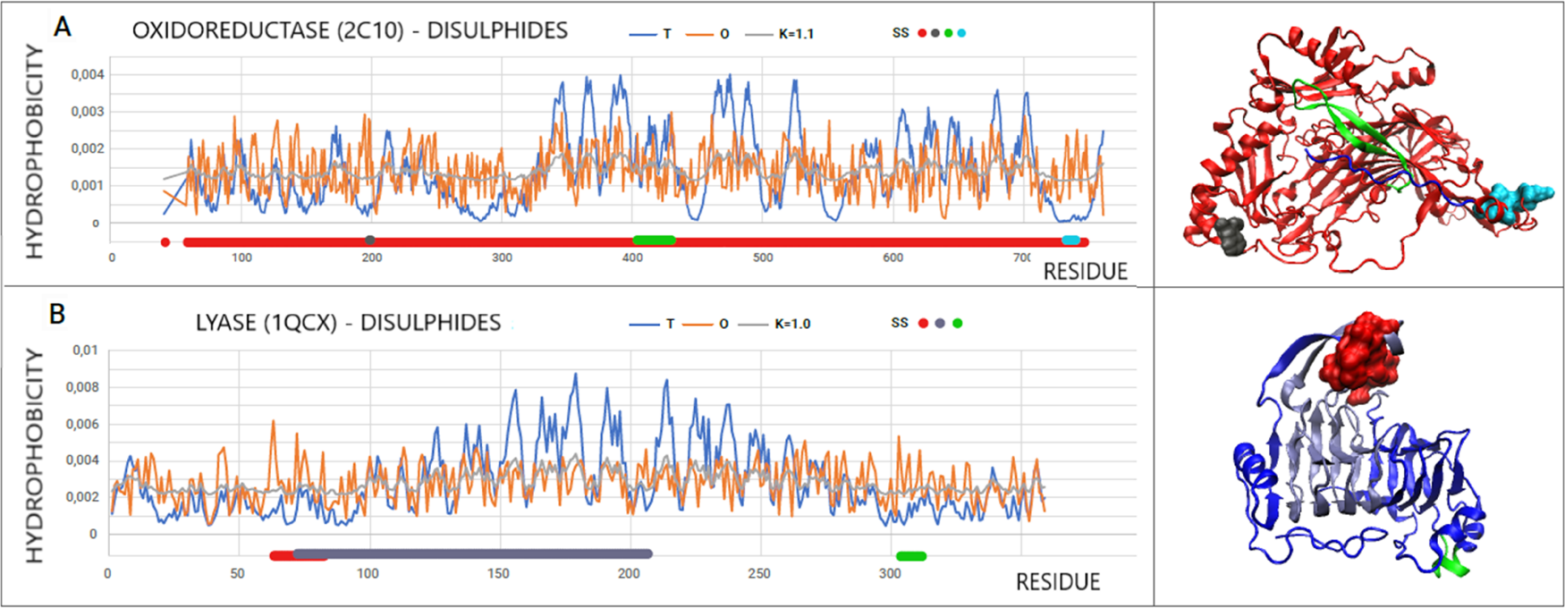
Arrangement of disulfide bonds and their arrangement in the 3D structure. (A) Oxidoreductase (PDB ID 2C10): magenta and gray section—space-filling presentation. (B) Lyase (PDB ID 1QCX): section 63–82 was distinguished as space-filling because it is partly included in the binding section SS 72–206. Fragments in accordance with the colors of the lines on the profiles.

In the case of oxidoreductase (PDB ID 2C10), the disulfide bond (41–748) covers almost the entire chain connecting the N- and C-terminal positions. Therefore, it is not surprising that the values of RD and *K* are very similar to those of the entire chain (Figure 11A). Dom3, characterized by high RD and *K* values, contains a section marked with a disulfide bond with a status showing local micelle-like order, although the length of this fragment is not large. The C-terminal segment not being part of any domain shows a status comparable to that of the entire chain. Note the stabilization of sections locally showing an excess of hydrophobicity in the positions of disulfides 198–199 and 734–741 (Figure 11A)

In the system of disulfide bonds in lyase (PDB ID 1QCX), the 72–206 bond is distinguished, covering a significant part of the chain and showing a status comparable to that of the entire molecule. A short section (303–311) shows a locally close micelle-like order. The intermediate status—albeit close to the status of the entire chain—is shown by section 63–82 (Figure 11B). In this protein, similar to the previous one, sections limited by disulfide bonds show a local excess of hydrophobicity 63–82 and 303–311 (Figure 11B).

There are two types of disulfide bond analysis based on the fuzzy oil drop model: (1). The arrangement of disulfide bonds interacts with the micelle-like arrangement. (2). The system of disulfide bonds introduces a status different from the expected micelle-like type.^50^ The role of the first type is defined as a concerted action to stabilize the structure, while the second type represents the presence of an agent stabilizing the nonmicelle-like system. In both cases, the presence of disulfide bonds can be considered as a component of this factor, which is expressed in the *K* parameter. In addition to the influence of the water environment modified by the presence of other components, including a completely different field, such as the membrane, the system of disulfide bonds may play a significant role in the folding process.

## 4 Discussion

The polar water environment directs the structuring of bipolar molecules into the form of micelles with a centric concentration of hydrophobicity with a polar outer shell, ensuring entropically favorable contact with the environment. Bipolar amino acids reproduce the structure of micelles to a limited extent, depending on the ability of the amino acid sequence to achieve such an order. These conditions are changed by factors such as the amphipathic membrane or the specificity of the periplasmic environment.^51^ The diversity resulting from the variability of amino acid sequences is complemented not only by the diversity of the environment in the form of an external force field provided by the changed water characteristics (pH, ionic strength) but also by the presence of other molecules affecting the folding process—prefoldin,^52^ chaperones, or chaperonins.^53,54^ These molecules act periodically to support the folding process at a specific stage. Obtaining a significant reduction in the energy barrier, which takes place in the case of enzymatic catalysis, results from the specific arrangement of catalytic residues and the surrounding force field. The large size of enzymes or receptors probably results from the need to introduce an appropriate environment that provides an appropriate, specific distribution of forces. The part outside the catalytic center seems to provide the right conditions in the form of an external force field. In the discussion on enzymatic activity, all kinds of interactions are carefully considered, including the nonbinding interactions. Their drawback lies in the rather advanced level of detail expressed in the form of atom–atom interaction or even on the electron–electron level (QM/MM method^55−57^). Hydrophobic interactions are outside the scope of this detailed interpretation. According to the hypothesis proposed in the present work, it was suggested to construct the force field on the basis of the hydrophobicity dispersion, the variability and diversity of which provide conditions for very diverse processes. The protein body in the examples discussed here is nothing more than a permanent chaperone, which ensures the stability of the specificity of the environment of the molecule, even with the variability of external conditions. A suitable system different from that imposed by the aqueous environment seems to play a critical role in biological activity.

Designing de novo enzymes with a given expected activity should take into account not only the construction of the active group itself but also the local constant environment, which, regardless of the variability of, for example, a specific temperature range or even pH, can ensure the stability of the field constituting the base for the process. This role is probably played by all nonprotein components, including the ions also listed for primary amine oxidase (2C10).^11−13,58−66^ Noncompetitive inhibitors bound in a location distant from the active center yet inhibiting the course of the reaction may be an example of a force field disorder, which, by not providing its appropriate version, inhibits the course of the reaction.

As long as attention is focused on the building components in the sense of secondary structure, the planned, expected activity of enzymes is not achieved.^67^

Studies focused on enzymatic activity consider the importance of the electrostatic field.^68−71^ Nonbinding interactions: electrostatic and vdW examined with the FOD model in search of possible differentiation of the distribution of this type of interactions indicate their R type dispersion, which is uniform. Optimization methods (e.g., in the process of protein structure prediction) turn out to be appropriate in this context. The need for any direction of these effects has not been identified.^72,73^

The set of enzymes presented is limited to a few examples. Large-scale analysis is needed and will continue. Nevertheless, the diversity of the discussed examples confirms the hypothesis about the need for the presence of an external force field that is appropriate for a given enzyme.

The proteins discussed in detail here are supplemented by a list of proteins—enzymes with similar characteristics (Supporting Information).

The alternative analysis of enzymatic catalysis is presented in ref (74). Authors focus the attention on the relation between very high variability to running many specific reactions using quite limited tools such as only few amino acids. The analysis covers the whole spectrum from large-scale general statistics to individual analysis of the chemical specificity of residues participating in reactions.^75,76^ The analysis is focused on the catalytic center and the specific role of catalytic residues. The model presented in this paper can be treated as supplementary as taking into account the close neighborhood delivering the local external force field for catalytic residues. The presented model is even more complementary to the one distinguishing “passive” and “intrusive” mechanisms, suggesting two steps of the catalytic reaction: scanning and the act of catalysis.^77^ The status of the local surroundings of catalytic residues is the method to send the signal to the environment, ordering water molecules in the appropriate form recognized by the substrate molecule. The influence of mutations in evolutionary history is treated as a source of information consumed in de novo design enzymes.^78^ The explanation of the activity of cold-adapted enzymes (25–0 °C) is the real challenge, especially taking into account the influence of salt concentration on their activity.^79^ The hypothesis assuming the role of transition state distinguishing “positive” and “negative” catalysis suggests the participation of positive and negative constraints in the catalytic landscape of enzymes.^80^

Extensively developed techniques based on artificial intelligence adopting machine learning are expected to help in the de novo design of enzymes potentially applied in therapy.^81,82^ Nonaqueous enzymology seems to be of high interest in the context of the presented model, where the environment is treated as an important component conditioning the enzyme activity.^83^

Participation of the environment influencing the inter-residual interaction generating the appropriate secondary structure in water-soluble as well as membrane proteins, especially their helical forms, is discussed in detail in ref (84,85).

Treating all mentioned methods together with the model proposed in this paper may deliver an effective tool for the de novo design of enzymes of expected, defined activity.^86^

## 5 Summary

In the present work, different constructions of the field surrounding the environment of the enzymatic reaction have been revealed. This diversity covers a wide spectrum in the light of parameters based on the fuzzy oil drop model—from structures resulting from the influence of the aqueous environment (low RD and *K* values close to zero), where the protein structure reproduces a micelle-like structure, through structures of this type with local disorder in the immediate vicinity catalytic site (RD slightly above 0.5 with residues with an excess/deficit of hydrophobicity located in specific sections of the chain) to complex structures with high RD (RD > 0.7) and *K* (*K* ≥ 1.0) values. In the case of the latter, it is possible to determine the characteristics of the external force field constituting the base for the catalytic reaction, first of all, by isolating it from the influence of the water environment with the simultaneous imposition of a specific environment for the catalytic reaction. The question of how proteins with high RD are folded remains an open question. Such an analysis is currently underway.^53,54^

Conclusions resulting from the obtained results reveal the need for a new approach to the de novo design of enzymes. It is not enough to build a system of catalytic residues. The environment provided by the protein for a given reaction is important. Traditionally, the identification of the so-called active site comes down to the mutual arrangement of catalytic residues. It turns out, however, that these residues are able to perform a reaction with the active participation of the entire molecule, which creates appropriate environmental conditions. In the case of a comparative analysis of isomerases,^84^ the local deviation from the micelle-like system also includes residues not identified as catalytic residues (directly involved in the course of the reaction). The demonstration that these residues introduce a local excess of hydrophobicity in the immediate vicinity of the catalytic center can be treated as an active participation in the catalytic capabilities by targeting and perhaps recognizing, and certainly a specific type of sending information to the environment addressed to the potential substrate.^20^

The approach presented in this paper discusses the force field generated by the whole enzyme molecule, in contrast to many other models discussing the enzymatic cavity in form limited and focused solely on catalytic residues.

## Supporting Information Available

List of 265 enzymes representing the ECs (enzymatic classes) characterized by the values of RD and *K* parameters to support the results described in detail in the paper (PDF)

## References

[ref1] Richard J. P. Enabling Role of Ligand-Driven Conformational Changes in Enzyme Evolution. Biochemistry 2022, 61 (15), 1533–1542. 10.1021/acs.biochem.2c00178. 35829700 PMC9354746

[ref2] Wolfenden R. Thermodynamic and extrathermodynamic requirements of enzyme catalysis. Biophys. Chem. 2003, 105 (2–3), 559–572. 10.1016/S0301-4622(03)00066-8. 14499918

[ref3] Morrow J. R.; Amyes T. L.; Richard J. P. Phosphate binding energy and catalysis by small and large molecules. Acc. Chem. Res. 2008, 41 (4), 539–548. 10.1021/ar7002013. 18293941 PMC2652674

[ref4] Amyes T. L.; Richard J. P. Specificity in transition state binding: the Pauling model revisited. Biochemistry 2013, 52 (12), 2021–2035. 10.1021/bi301491r. 23327224 PMC3679207

[ref5] Wolfenden R.; Snider M. J. The depth of chemical time and the power of enzymes as catalysts. Acc. Chem. Res. 2001, 34 (12), 938–945. 10.1021/ar000058i. 11747411

[ref6] O’Sullivan J.; Unzeta M.; Healy J.; O’Sullivan M. I.; Davey G.; Tipton K. F. Semicarbazide-sensitive amine oxidases: enzymes with quite a lot to do. Neurotoxicology 2004, 25 (1–2), 303–315. 10.1016/S0161-813X(03)00117-7. 14697905

[ref7] Abel R.; Young T.; Farid R.; Berne B. J.; Friesner R. A. Role of the active-site solvent in the thermodynamics of factor Xa ligand binding. J. Am. Chem. Soc. 2008, 130 (9), 2817–2831. 10.1021/ja0771033. 18266362 PMC2761766

[ref8] Pauling L. Molecular Architecture and Biological Reactions. Chem. Eng. News Arch. 1946, 24, 1375–1377. 10.1021/cen-v024n010.p1375.

[ref9] Nagel Z. D.; Klinman J. A 21st century revisionist’s view at a turning point in enzymology. Nat. Chem. Biol. 2009, 5, 543–550. 10.1038/nchembio.204. 19620995

[ref10] Whitesides G. M. Applications of Cell-Free Enzymes in Organic Synthesis. In Ciba Foundation Symposium 111 - Enzymes in Organic Synthesis; Wiley, 1985; Vol. 111, pp 76–96. 10.1002/9780470720929.ch7 3848381

[ref11] Kries H.; Blomberg R.; Hilvert D. De novo enzymes by computational design. Curr. Opin. Chem. Biol. 2013, 17 (2), 221–228. 10.1016/j.cbpa.2013.02.012. 23498973

[ref12] Vaissier Welborn V.; Head-Gordon T. Computational Design of Synthetic Enzymes. Chem. Rev. 2019, 119 (11), 6613–6630. 10.1021/acs.chemrev.8b00399. 30277066

[ref13] Fuxreiter M.; Mones L. The role of reorganization energy in rational enzyme design. Curr. Opin. Chem. Biol. 2014, 21, 34–41. 10.1016/j.cbpa.2014.03.011. 24769299

[ref14] Kaplan J.; DeGrado W. F. De novo design of catalytic proteins. Proc. Natl. Acad. Sci. U.S.A. 2004, 101 (32), 11566–11570. 10.1073/pnas.0404387101. 15292507 PMC511021

[ref15] Turner A. J.; Fisk L.; Nalivaeva N. N. Targeting amyloid-degrading enzymes as therapeutic strategies in neurodegeneration. Ann. N. Y. Acad. Sci. 2004, 1035, 1–20. 10.1196/annals.1332.001. 15681797

[ref16] Carson J. A.; Turner A. J. Beta-amyloid catabolism: roles for neprilysin (NEP) and other metallopeptidases?. J. Neurochem. 2002, 81 (1), 1–8. 10.1046/j.1471-4159.2002.00855.x. 12067222

[ref17] Nalivaeva N. N.; Fisk L. R.; Belyaev N. D.; Turner A. J. Amyloid-degrading enzymes as therapeutic targets in Alzheimer’s disease. Curr. Alzheimer Res. 2008, 5 (2), 212–224. 10.2174/156720508783954785. 18393806

[ref18] Cruz-Vicente P.; Passarinha L. A.; Silvestre S.; Gallardo E. Recent Developments in New Therapeutic Agents against Alzheimer and Parkinson Diseases: In-Silico Approaches. Molecules 2021, 26 (8), 2193 10.3390/molecules26082193. 33920326 PMC8069930

[ref19] Banach M.; Stapor K.; Konieczny L.; Fabian P.; Roterman I. Downhill, Ultrafast and Fast Folding Proteins Revised. Int. J. Mol. Sci. 2020, 21 (20), 7632 10.3390/ijms21207632. 33076540 PMC7589632

[ref20] Roterman I.; Konieczny L. Protein is the inteligent micelle. Entropy 2023, 25 (6), 850 10.3390/e25060850. 37372194 PMC10297380

[ref21] Liu Y.; Beveridge D. L. Exploratory studies of ab initio protein structure prediction: multiple copy simulated annealing, AMBER energy functions, and a generalized born/solvent accessibility solvation model. Proteins 2002, 46 (1), 128–146. 10.1002/prot.10020. 11746709

[ref22] Grosdidier A.; Zoete V.; Michielin O. Fast docking using the CHARMM force field with EADock DSS. J. Comput. Chem. 2011, 32 (10), 2149–2159. 10.1002/jcc.21797. 21541955

[ref23] Roterman I. K.; Lambert M. H.; Gibson K. D.; Scheraga H. A. A comparison of the CHARMM, AMBER and ECEPP potentials for peptides. II. Phi-psi maps for N-acetyl alanine N’-methyl amide: comparisons, contrasts and simple experimental tests. J. Biomol. Struct. Dyn. 1989, 7 (3), 421–453. 10.1080/07391102.1989.10508503. 2627294

[ref24] Musah R. A.; Jensen G. M.; Bunte S. W.; Rosenfeld R. J.; Goodin D. B. Artificial protein cavities as specific ligand-binding templates: characterization of an engineered heterocyclic cation-binding site that preserves the evolved specificity of the parent protein. J. Mol. Biol. 2002, 315 (4), 845–857. 10.1006/jmbi.2001.5287. 11812152

[ref25] Jakobsson E.; Nilsson J.; Ogg D.; Kleywegt G. J. Structure of human semicarbazide-sensitive amine oxidase/vascular adhesion protein-1. Acta Crystallogr., Sect. D: Biol. Crystallogr. 2005, 61, 1550–1562. 10.1107/S0907444905028805. 16239734

[ref26] Mao C.; Cook W. J.; Zhou M.; Federov A. A.; Almo S. C.; Ealick S. E. Calf spleen purine nucleoside phosphorylase complexed with substrates and substrate analogues. Biochemistry 1998, 37 (20), 7135–7146. 10.1021/bi9723919. 9585525

[ref27] Wind R. D.; Uitdehaag J. C.; Buitelaar R. M.; Dijkstra B. W.; Dijkhuizen L. Engineering of cyclodextrin product specificity and pH optima of the thermostable cyclodextrin glycosyltransferase from Thermoanaerobacterium thermosulfurigenes EM1. J. Biol. Chem. 1998, 273 (10), 5771–5779. 10.1074/jbc.273.10.5771. 9488711

[ref28] Wang Z.; Quiocho F. A. Complexes of adenosine deaminase with two potent inhibitors: X-ray structures in four independent molecules at pH of maximum activity. Biochemistry 1998, 37 (23), 8314–8324. 10.1021/bi980324o. 9622483

[ref29] Vitali J.; Schick B.; Kester H. C.; Visser J.; Jurnak F. The tree-dimensional structure of aspergillus niger pectin lyase B at 1.7-A resolution. Plant Physiol. 1998, 116 (1), 69–80. 10.1104/pp.116.1.69. 9449837 PMC35189

[ref30] Verlinde C. L.; Noble M. E.; Kalk K. H.; Groendijk H.; Wierenga R. K.; Hol W. G. Anion binding at the active site of trypanosomal triosephosphate isomerase. Monohydrogen phosphate does not mimic sulphate. Eur. J. Biochem. 1991, 198 (1), 53–57. 10.1111/j.1432-1033.1991.tb15985.x. 2040290

[ref31] Käck H.; Gibson K. J.; Lindqvist Y.; Schneider G. Snapshot of a phosphorylated substrate intermediate by kinetic crystallography. Proc. Natl. Acad. Sci. U.S.A. 1998, 95 (10), 5495–5500. 10.1073/pnas.95.10.5495. 9576910 PMC20405

[ref32] Konieczny L.; Roterman I. Description of Fuzzy Oil Drop Model. In From Globular Proteins to Amyloids; Elsevier: Amsterdam Netherlands, Oxford UK, Cambridge MA USA, 2020; pp 1–11.

[ref33] Konieczny L.; Brylinski M.; Roterman I. Gauss function-based model of hydrophobicity density in proteins. In Silico Biol. 2006, 6, 15–22. 16789910

[ref34] Levitt M. A. A simplified representation of protein conformations for rapid simulation of protein folding. J. Mol. Biol. 1976, 104 (1), 59–107. 10.1016/0022-2836(76)90004-8. 957439

[ref35] Kullback S.; Leibler R. A. On information and sufficiency. Ann. Math. Stat. 1951, 22 (1), 79–86. 10.1214/aoms/1177729694.

[ref36] Sałapa K.; Kalinowska B.; Jadczyk T.; Roterman I. Measurement of Hydrophobicity Distribution in Proteins – Non-redundant Protein Data Bank. Bio-Algorithms Med. Syst. 2012, 8, 327–338. 10.2478/bams-2012-0023.

[ref37] Kalinowska B.; Banach M.; Konieczny L.; Roterman I. Application of Divergence Entropy to Characterize the Structure of the Hydrophobic Core in DNA Interacting Proteins. Entropy 2015, 17 (3), 1477–1507. 10.3390/e17031477.

[ref38] Banach M.; Fabian P.; Stapor K.; Konieczny L.; Roterman I. Structure of the Hydrophobic Core Determines the 3D Protein Structure – Verification by Single Mutation Proteins. Biomolecules 2020, 10 (5), 767 10.3390/biom10050767. 32423068 PMC7281683

[ref39] Roterman I.; Stapor K.; Fabian P.; Konieczny L. The Functional Significance of Hydrophobic Residue Distribution in Bacterial Beta-Barrel Transmembrane Proteins. Membranes 2021, 11 (8), 580 10.3390/membranes11080580. 34436343 PMC8399255

[ref40] Roterman I.; Stapor K.; Fabian P.; Konieczny L.; Banach M. Model of Environmental Membrane Field for Transmembrane Proteins. Int. J. Mol. Sci. 2021, 22 (7), 3619 10.3390/ijms22073619. 33807215 PMC8036355

[ref41] Roterman I.; Stapor K.; Gądek K.; Gubała T.; Nowakowski P.; Fabian P.; Konieczny L. Dependence of Protein Structure on Environment: FOD Model Applied to Membrane Proteins. Membranes 2022, 12, 50 10.3390/membranes12010050. PMC8778870 35054576

[ref42] Roterman I.; Stapor K.; Fabian P.; Konieczny L. Connexins and Pannexins—Similarities and Differences According to the FOD-M Model. Biomedicines 2022, 10, 1504 10.3390/biomedicines10071504. 35884807 PMC9313468

[ref43] https://www.ks.uiuc.edu/Research/vmd/. (accessed Dec, 2022).

[ref44] Humphrey W.; Dalke A.; Schulten K. VMD – Visual Molecular Dynamics. J. Mol. Graphics 1996, 14, 33–38. 10.1016/0263-7855(96)00018-5. 8744570

[ref45] Dygut J.; Kalinowska B.; Banach M.; Piwowar M.; Konieczny L.; Roterman I. Structural Interface Forms and Their Involvement in Stabilization of Multidomain Proteins or Protein Complexes. Int. J. Mol. Sci. 2016, 17 (10), 1741 10.3390/ijms17101741. 27763556 PMC5085769

[ref46] Banach M.; Roterman I. Solenoid—An Amyloid undr Control. In From Globular Proteins to Amyloids; Elsevier: Amsterdam Netherlands, 2020; pp 95–115.

[ref47] Tipton K. F.; O’Sullivan M. I.; Davey G. P.; O’Sullivan J. It can be a complicated life being an enzyme. Biochem. Soc. Trans. 2003, 31, 711–715. 10.1042/bst0310711. 12773189

[ref48] Dunkel P.; Balogh B.; Meleddu R.; Maccioni E.; Gyires K.; Mátyus P. Semicarbazide-sensitive amine oxidase/vascular adhesion protein-1: a patent survey. Expert Opin. Ther. Pat. 2011, 21 (9), 1453–1471. 10.1517/13543776.2011.594040. 21675926

[ref49] Brazeau B. J.; Johnson B. J.; Wilmot C. M. Copper-containing amine oxidases. Biogenesis and catalysis; a structural perspective. Arch. Biochem. Biophys. 2004, 428 (1), 22–31. 10.1016/j.abb.2004.03.034. 15234266

[ref50] Banach M.; Kalinowska B.; Konieczny L.; Roterman I. Role of Disulfide Bonds in Stabilizing the Conformation of Selected Enzymes—An Approach Based on Divergence Entropy Applied to the Structure of Hydrophobic Core in Proteins. Entropy 2016, 18 (3), 67 10.3390/e18030067.

[ref51] Roterman I.; Sieradzan A.; Stapor K.; Fabian P.; Wesołowski P.; Konieczny L. On the need to introduce environmental characteristics in ab initio protein structure prediction using a coarse-grained UNRES force field. J. Mol. Graphics Modell. 2022, 114, 108166 10.1016/j.jmgm.2022.108166. 35325843

[ref52] Roterman I.; Konieczny L. Model of external force field for protein folding process – role of prefoldin – submitted. 10.3389/fchem.2024.1342434 PMC11002104 38595701

[ref53] Roterman I.; Stapor K.; Konieczny L. Ab initio protein structure prediction: the necessary presence of external force field as it is delivered by Hsp40 chaperone. BMC Bioinf. 2023, 24 (1), 418 10.1186/s12859-023-05545-0. PMC10629080 37932669

[ref54] Roterman I.; Stapor K.; Dulak D.; Konieczny L. External force field for protein folding in chaperonines – potential application for In Silico protein folding – submitted. 10.1021/acsomega.4c00409 PMC11044213 38680295

[ref55] Ryde U. QM/MM Calculations on Proteins. In Methods in Enzymology; Elsevier, 2016; Vol. 577, pp 119–158. 27498637 10.1016/bs.mie.2016.05.014

[ref56] Cui Q.; Pal T.; Xie L. Biomolecular QM/MM Simulations: What Are Some of the ″Burning Issues″?. J. Phys. Chem. B 2021, 125 (3), 689–702. 10.1021/acs.jpcb.0c09898. 33401903 PMC8360698

[ref57] Senn H. M.; Thiel W. QM/MM studies of enzymes. Curr. Opin. Chem. Biol. 2007, 11 (2), 182–187. 10.1016/j.cbpa.2007.01.684. 17307018

[ref58] Lipsh-Sokolik R.; Khersonsky O.; Schröder S. P.; de Boer C.; Hoch S.-Y.; Davies G. J.; Overkleeft H. S.; Fleishman S. J. Combinatorial assembly and design of enzymes. Science 2023, 379 (6628), 195–201. 10.1126/science.ade9434. 36634164

[ref59] Csete M. E.; Doyle J. C. Reverse engineering of biological complexity. Science 2002, 295 (5560), 1664–1669. 10.1126/science.1069981. 11872830

[ref60] Rocklin G. J.; Chidyausiku T. M.; Goreshnik I.; Ford A.; Houliston S.; Lemak A.; Carter L.; Ravichandran R.; Mulligan V. K.; Chevalier A.; Arrowsmith C. H.; Baker D. Global analysis of protein folding using massively parallel design, synthesis, and testing. Science 2017, 357 (6347), 168–175. 10.1126/science.aan0693. 28706065 PMC5568797

[ref61] Hilvert D. Design of protein catalysts. Annu. Rev. Biochem. 2013, 82, 447–470. 10.1146/annurev-biochem-072611-101825. 23746259

[ref62] Kaushik M.; Sinha P.; Jaiswal P.; Mahendru S.; Roy K.; Kukreti S. Protein engineering and de novo designing of a biocatalyst. J. Mol. Recognit. 2016, 29 (10), 499–503. 10.1002/jmr.2546. 27113645

[ref63] Bolon D. N.; Voigt C. A.; Mayo S. L. De novo design of biocatalysts. Curr. Opin. Chem. Biol. 2002, 6 (2), 125–129. 10.1016/S1367-5931(02)00303-4. 12038994

[ref64] Richter F.; Leaver-Fay A.; Khare S. D.; Bjelic S.; Baker D. De novo enzyme design using Rosetta3. PLoS One 2011, 6 (5), e19230 10.1371/journal.pone.0019230. 21603656 PMC3095599

[ref65] Kaplan J.; DeGrado W. F. De novo enzyme design using Rosetta3. Proc. Natl. Acad. Sci. U.S.A. 2004, 101 (32), 11566–11570. 10.1073/pnas.0404387101. 15292507 PMC511021

[ref66] Lipsh-Sokolik R.; Khersonsky O.; Schröder S. P.; de Boer C.; Hoch S. Y.; Davies G. J.; Overkleeft H. S.; Fleishman S. J. Combinatorial assembly and design of enzymes. Science 2023, 379 (6628), 195–201. 10.1126/science.ade9434. 36634164

[ref67] Kim T.-E.; Tsuboyama K.; Houliston S.; Martell C. M.; Phoumyvong C. M.; Lemak A.; Haddox H. K.; Arrowsmith C. H.; Rocklin G. J. Dissecting the stability determinants of a challenging de novo protein fold using massively parallel design and experimentation. Proc. Natl. Acad. Sci. U.S.A. 2022, 119 (41), e2122676119 10.1073/pnas.2122676119. 36191185 PMC9564214

[ref68] Fried S. D.; Boxer S. G. Electric Fields and Enzyme Catalysis. Annu. Rev. Biochem. 2017, 86, 387–415. 10.1146/annurev-biochem-061516-044432. 28375745 PMC5600505

[ref69] Wu Y.; Boxer S. G. A Critical Test of the Electrostatic Contribution to Catalysis with Noncanonical Amino Acids in Ketosteroid Isomerase. J. Am. Chem. Soc. 2016, 138 (36), 11890–11895. 14 10.1021/jacs.6b06843. 27545569 PMC5063566

[ref70] Wang X.; He X.; Zhang J. Z. Accurate Calculation of Electric Fields Inside Enzymes. In Methods in Enzymology; Elsevier, 2016; Vol. 578, pp 45–72. 27497162 10.1016/bs.mie.2016.05.043

[ref71] Hammes-Schiffer S. Catalytic efficiency of enzymes: a theoretical analysis. Biochemistry 2013, 52 (12), 2012–2020. 10.1021/bi301515j. 23240765 PMC3619019

[ref72] Ptak-Kaczor M.; Banach M.; Konieczny L.; Roterman I. Internal force field in selected proteins Acta Biochim. Pol. 2019; Vol. 66 (4), 10.18388/abp.2019_2865. 31747510

[ref73] Marchewka D.; Banach M.; Roterman I. Internal force field in proteins seen by divergence entropy. Bioinformation 2011, 6 (8), 300–302. 10.6026/97320630006300. 21769190 PMC3134777

[ref74] Ribeiro A. J. M.; Tyzack J. D.; Borkakoti N.; Holliday G. L.; Thornton J. M. A global analysis of function and conservation of catalytic residues in enzymes. J. Biol. Chem. 2020, 295 (2), 314–324. 10.1074/jbc.REV119.006289. 31796628 PMC6956550

[ref75] Ribeiro A. J. M.; Holliday G. L.; Furnham N.; Tyzack J. D.; Ferris K.; Thornton J. M. Mechanism and Catalytic Site Atlas (M-CSA): a database of enzyme reaction mechanisms and active sites. Nucleic Acids Res. 2018, 46, D618–D623. 10.1093/nar/gkx1012. 29106569 PMC5753290

[ref76] https://www.ebi.ac.uk/thornton-srv/m-csa/.

[ref77] Mak C. H.; Pham P.; Afif S. A.; Goodman M. F. Random-walk enzymes. Phys. Rev. E 2015, 92 (3), 032717 10.1103/PhysRevE.92.032717. PMC4672870 26465508

[ref78] Bunzel H. A.; Anderson J. L. R.; Mulholland A. J. Designing better enzymes: Insights from directed evolution. Curr. Opin. Struct. Biol. 2021, 67, 212–218. 10.1016/j.sbi.2020.12.015. 33517098

[ref79] Liu Y.; Jia K.; Chen H.; Wang Z.; Zhao W.; Zhu L. Cold-adapted enzymes: mechanisms, engineering and biotechnological application. Bioprocess Biosyst. Eng. 2023, 46 (10), 1399–1410. 10.1007/s00449-023-02904-2. 37486422

[ref80] Vögeli B.; Erb T. J. Negative’ and ’positive catalysis’: complementary principles that shape the catalytic landscape of enzymes. Curr. Opin. Chem. Biol. 2018, 47, 94–100. 10.1016/j.cbpa.2018.09.013. 30268906

[ref81] Planas-Iglesias J.; Marques S. M.; Pinto G. P.; Musil M.; Stourac J.; Damborsky J.; Bednar D. Computational design of enzymes for biotechnological applications. Biotechnol. Adv. 2021, 47, 107696 10.1016/j.biotechadv.2021.107696. 33513434

[ref82] Vasina M.; Velecký J.; Planas-Iglesias J.; Marques S. M.; Skarupova J.; Damborsky J.; Bednar D.; Mazurenko S.; Prokop Z. Tools for computational design and high-throughput screening of therapeutic enzymes. Adv. Drug Delivery Rev. 2022, 183, 114143 10.1016/j.addr.2022.114143. 35167900

[ref83] Kikani B.; Patel R.; Thumar J.; Bhatt H.; Rathore D. S.; Koladiya G. A.; Singh S. P. Solvent tolerant enzymes in extremophiles: Adaptations and applications. Int. J. Biol. Macromol. 2023, 238, 124051 10.1016/j.ijbiomac.2023.124051. 36933597

[ref84] Jha A. N.; Vishveshwara S.; Banavar J. R. Amino acid interaction preferences in proteins. Protein Sci. 2010, 19 (3), 603–616. 10.1002/pro.339. 20073083 PMC2866284

[ref85] Jha A. N.; Vishveshwara S.; Banavar J. R. Amino acid interaction preferences in helical membrane proteins. Protein Eng., Des. Sel. 2011, 24 (8), 579–588. 10.1093/protein/gzr022. 21666247

[ref86] Roterman I.; Stapor K.; Konieczny L. New insights on the catalytic center of proteins from peptidylprolyl isomerase group. J. Cell. Biochem. 2023, 124 (6), 818–835. 10.1002/jcb.30407. 37139783

