# Supplementary material for: Hydrophobicity-Based Force Field In Enzymes
Source: ACS Omega. 2024 Feb 7;9(7):8188–203. doi: 10.1021/acsomega.3c08728 (PMC10882594; doi:10.1021/acsomega.3c08728)
Supplement: Supplementary file 1 — ao3c08728_si_001.pdf [file ao3c08728_si_001.pdf]

SUPPLEMENTARY MATERIALS

HYDROPHOBICITY-BASED FORCE FIELD IN ENZYMES

Irena Roterman [1,*] , Leszek Konieczny [2] Katarzyna Stapor[3] and Mateusz Słupina [4]

1 Department of Bioinformatics and Telemedicine, Jagiellonian University – Medical College, 30-688 Kraków Medyczna 7, Poland myroterm@cyf-kr.edu.pl

2 Chair of Medical Biochemistry, Jagiellonian University – Medical College, 31-034 Kraków, Kopernika 7 Poland mbkoniec@cyf-kr.edu.pl

3 Faculty of Automatic, Electronics and Computer Science, Department of Applied Informatics, Silesian University of Technology, Akademicka 16, 44-100 Gliwice, Poland, katarzyna.stapor@polsl.pl (K.S.)

4 ALSTOM ZWUS Sp. z o.o. Modelarska 12, 40-142 Katowice, mateusz.slupina@wp.pl

* Correspondence: myroterm@cyf-kr.edu.pl

PDB ID	E CLASS	RD	K
**E.C.1.**		**25.7%**	
3TYW-„C”	E.C.1.	0.601	0.75
3TVZ-„C”	E.C.1.	0.416	0.26
1FP4-„D”	E.C.1.	0.625	0.74
2H9A-„D”	E.C.1.	0.435	0.25
4C9P-„D”	E.C.1.	0.591	0.65
1Y6D	E.C.1.	0.556	0.47
3RFR-„C”	E.C.1.	0.622	0.48
3I4F-„C”	E.C.1.	0.502	0.42
3E39-„D”	E.C.1.	0.438	0.30
2RE2-„D”	E.C.1.	0.419	0.27
2Q0L-„D”	E.C.1.	0.416	0.24
3CNT	E.C.1.	0.471	0.33
4IBO-„C”	E.C.1.	0.539	0.46
3DQP	E.C.1.	0.521	0.50
1ZEM-„C”	E.C.1.1.1.9	0.589	0.63
2E37-„C”	E.C.1.1.1.27	0.714	0.52
1WDK-„C”	E.C.1.1.1.35	0.492	0.40
3BLX-„C”	E.C.1.1.1.41	0.667	0.8
2ZYD-„D”	E.C.1.1.1.44	0.640	0.72
2AYQ-„D”	E.C.1.1.1.85	0.609	0.71
4DMM-„C”	E.C.1.1.1.100	0.558	0.51
3FTP-„C”	E.C.1.1.1.100	0.577	0.54
2NM0-„D”	E.C.1.1.1.100	0.501	0.43
1ULS-„C”	E.C.1.1.1.100	0.532	0.46
4GKB-„C”	E.C.1.1.1.100	0.550	0.46

3VTZ-„C”	E.C.1.1.1.118	0.445	0.24
3UF0-„D”	E.C.1.1.1.125	0.603	0.60
4BB6-„D”	E.C.1.1.1.146	0.546	0.45
3DWF-„D”	E.C.1.1.1.146	0.589	0.57
1LRT-„C”	E.C.1.1.1.205	0.524	0.41
1AE1-„D”	E.C.1.1.1.206	0.468	0.35
3TSN-„D”	E.C.1.1.1.262	0.624	0.81
1XG5-„C”	E.C.1.1.1.270	0.519	0.46
1GEG-„C”	E.C.1.1.1.304	0.529	0.46
1A80	E.C.1.1.1.346	0.686	1.04
4M7S	E.C.1.1.99.38	0.513	0.49
3HAZ-„D”	E.C.1.2.1.88	0.715	1.27
1W85-„C”	E.C.1.2.4.1	0.660	0.97
1UMD-„C”	E.C.1.2.4.4	0.660	0.94
2C42-„C”	E.C.1.2.7.1	0.675	0.85
2AQI	E.C.1.3.1.9	0.621	0.72
3E8J-„D”	E.C.1.3.3.3	0.461	0.36
3HML-„D”	E.C.1.3.3.11	0.650	0.83
2YNM-„C”	E.C.1.3.7.7	0.475	0.34
1T9M-„D”	E.C.1.4	0.590	0.53
3RHA-„D”	E.C.1.4.3.10	0.613	0.74
3ROA-„D”	E.C.1.5.1.3	0.454	0.33
1E7W-„D”	E.C.1.5.1.33	0.541	0.45
3N2S-„D”	E.C.1.5.1.38	0.427	0.28
1ZCH	E.C.1.5.1.39	0.441	0.31
1Z69-„C”	E.C.1.5.98.2	0.611	0.78
1YDG-„C”	E.C.1.6.5.2	0.556	0.50
4N4J	E.C.1.7.2.8	0.689	0.99
1BHY	E.C.1.8.1.4	0.639	0.83
4J56-„C”	E.C.1.8.1.9	0.450	0.77
1QWL-„D”	E.C.1.11.1.6	0.438	0.69
2ZCT-„C”	E.C.1.11.1.24	0.611	0.67
1PSQ-„D”	E.C.1.11.1.24	0.534	0.52
1FRV-„C”	E.C.1.12.2.1	0.631	0.78
1FGQ	E.C.1.13.11.12	0.709	1.11
1CPT	E.C.1.14	0.617	0.73
4FFA-„C”	E.C.1.14.11	0.845	0.74
1PKF	E.C.1.14.15	0.500	0.43
1Z8P	E.C.1.14.15.36	0.591	0.71
4J5I-„C”	E.C.1.14.11.17	0.632	0.73
1JK0-„D”	E.C.1.17.4.1	0.487	0.39
**E.C.2.**		**33.3%**	
2R1I-„D”	E.C.2.	0.481	0.35
2DXQ	E.C.2.	0.515	0.42
3JWG	E.C.2.	0.414	0.32
3PKO-„D”	E.C.2.	0.600	0.71
3KO5	E.C.2.	0.339	0.16

3R7A-„D”	E.C.2.	0.589	0.65
2ZIE-„D”	E.C.2.1.1	0.488	0.35
4LEC-„D”	E.C.2.1.1	0.335	0.14
3GDH-„D”	E.C.2.1.1	0.556	0.55
3AV4	E.C.2.1.1.37	0.734	1.33
2QY2-„D”	E.C.2.1.1.56	0.590	0.69
2PLW	E.C.2.1.1.56	0.454	0.34
4A2N	E.C.2.1.1.100	0.589	0.68
1SUI	E.C.2.1.1.104	0.534	0.51
3N4J	E.C.2.1.1.207	0.426	0.27
2OGY-„D”	E.C.2.1.1.258	0.508	0.44
3OII-„D”	E.C.2.1.1.260	0.411	0.26
3S8S	E.C.2.1.1.354	0.520	0.43
1XJW	E.C.2.1.3.2	0.640	0.82
2F9I-„C”	E.C.2.1.3.15	0.533	0.36
1AY0-„D”	E.C.2.2.1.1	0.688	1.02
3OWC-„D”	E.C.2.3.1	0.479	0.40
1SCZ	E.C.2.3.1.61	0.461	0.25
2VAV-„C”	E.C.2.3.1.175	0.544	0.47
3A5S-„D”	E.C.2.3.1.212	0.536	0.52
2Y7E-„D”	E.C.2.3.1.247	0.509	0.48
3M7F-„D”	E.C.2.3.2.26	0.405	0.18
2P8U-„D”	E.C.2.3.3.10	0.564	0.57
1D3C	E.C.2.4.1.19	0.779	1.51
4CGT	E.C.2.4.1.19	0.787	1.57
1XFJ	E.C.2.4.2.1	0.468	0.39
2JBH-„D”	E.C.2.4.2.8	0.525	0.43
1X1O-„C”	E.C.2.4.2.19	0.712	1.03
1V4N-„C”	E.C.2.4.2.28	0.510	0.44
2Q80-„C”	E.C.2.5.1.1	0.640	0.93
4KKM-„D”	E.C.2.5.1.10	0.536	0.5
1EYE	E.C.2.5.1.15	0.608	0.60
3TBF-„C”	E.C.2.6.1.16	0.548	0.57
2COI-„D”	E.C.2.6.1.42	0.668	0.94
2RBC	E.C.2.7.1	0.432	0.26
3IKH-„C”	E.C.2.7.1.15	0.600	0.64
4I1U-„D”	E.C.2.7.1.24	0.701	1.07
1JXH-„D”	E.C.2.7.1.49	0.514	0.46
3DZV-„D”	E.C.2.7.1.50	0.413	0.29
1C3Q-„C”	E.C.2.7.1.50	0.457	0.33
3PNL-„D”	E.C.2.7.1.121	0.482	0.39
2018-„C”	E.C.2.7.1.180	0.593	0.57
4JKR-„C”	E.C.2.7.7.6	0.732	1.54
3IYD-„C”	E.C.2.7.7.6	0.771	1.75
1QKL	E.C.2.7.7.6	0.514	0.38
2HTF	E.C.2.7.7.7	0.491	0.33
2QH5	E.C.2.7.7.13	0.419	0.27
1KHV-„D”	E.C.2.7.7.48	0.665	1.15

3MMP	E.C.2.7.7.48	0.653	1.01
3VQ8-„D”	E.C.2.7.7.49	0.538	0.50
1P16	E.C.2.7.7.50	0.558	0.5
4J72-„D”	E.C.2.7.8.13	0.474	0.35
2KK1	E.C.2.7.10.2	0.532	0.42
1KKL-„C”	E.C.2.7.11	0.473	0.28
3RE4-„D”	E.C.2.7.11.1	0.491	0.39
1ZTH-„C”	E.C.2.7.11.1	0.564	0.63
4M66-„D”	E.C.2.7.11.1	0.476	0.54
3LCB-„C”	E.C.2.7.11.5	0.686	0.98
3LCB-„C”	E.C.2.7.11.5	0.686	0.98
3ENM-„C”	E.C.2.7.12.2	0.625	0.73
4EUK-„D”	E.C.2.7.13.3	0.416	0.28
1XVU	E.C.2.8.3.21	0.699	1.11
1MRO-„C”	E.C.2.8.4.1	0.618	0.63
**E.C.3.**		**23.7%**	
4J4J	E3	0.594	0.70
4BJF-„C”	E.C.3.	0.769	2.71
2Q78-„C”	E.C.3.	0.454	0.32
3RQZ-„C”	E.C.3.	0.538	0.49
3TM1-„D”	E.C.3.	0.513	0.37
3RXZ-„C”	E.C.3.	0.564	0.55
1UFO-„C”	E.C.3.	0.419	0.29
3QH4	E.C.3.	0.588	0.64
3DFI	E.C.3.	0.564	0.54
1J23	E.C.3.	0.514	0.46
3BBJ-„D”	E.C.3.	0.602	0.66
1T2W	E.C.3.	0.343	0.11
3PNZ-„C”	E.C.3.	0.532	0.57
3FCM-„D”	E.C.3.	0.466	0.36
3TM8-„D”	E.C.3.	0.607	0.43
3FV9-„C”	E.C.3.	0.673	1.03
2PR7	E.C.3.	0.476	0.38
4BGO-„D”	E.C.3.1	0.621	0.82
3H87-„D”	E.C.3.1	0.596	0.54
1CI9-„D”	E.C.3.1.1	0.535	0.69
1K4Y	E.C.3.1.1.1	0.566	0.60
2Q0S-„C”	E.C.3.1.1.2	0.602	0.67
1AKN	E.C.3.1.1.13	0.641	0.79
3HFQ-„D”	E.C.3.1.1.31	0.580	0.63
3HFQ	E.C.3.1.1.31	0.810	0.63
1NX9-„C”	E.C.3.1.1.43	0.632	0.75
1QOZ-„D”	E.C.3.1.1.72	0.663	0.79
3I2I	E.C.3.1.1.84	0.690	1.04
1VH9-„D”	E.C.3.1.2	0.517	0.46
4IJ5-„D”	E.C.3.1.3.3	0.481	0.42
2YXO-„D”	E.C.3.1.3.15	0.506	0.45

3N3C	E.C.3.1.3.43	0.567	0.52
1PTV	E.C.3.1.3.481	0.423	0.29
2DQB-„C”	E.C.3.1.5.1	0.614	0.7
1XWY	E.C.3.1.11	0.484	0.43
2X7V	E.C.3.1.21.2	0.520	0.53
1EYU-„D”	E.C.3.1.21.4	0.628	0.68
2WCZ-„D”	E.C.3.1.22.4	0.508	0.39
2UY2	E.C.3.2.1.14	0.609	0.73
1C10	E.C.3.2.1.17	0.585	0.56
3T1E-„D”	E.C.3.2.1.18	0.591	0.64
1Z4V	E.C.3.2.1.18	0.675	0.90
1TR1	E.C.3.2.1.21	0.574	0.62
1VFF	E.C.3.2.1.21	0.522	0.51
3T8J	E.C.3.2.2.1	0.489	0.41
2ZIC	E.C.3.2.1.70	0.617	0.76
1QNR	E.C.3.2.1.78	0.656	0.89
1M2X-„C”	E.C.3.2.6	0.330	0.11
3B3V	E.C.3.4.11.10	0.502	0.46
3GLJ	E.C.3.4.17.2	0.575	0.63
2QR5-„D”	E.C.3.4.19.1	0.626	0.82
4FGE	E.C.3.4.19.11	0.357	0.17
1XD3-„D”	E.C.3.4.19.12	0.406	0.27
2FMJ	E.C.3.4.21.4	0.480	0.39
1EZU-„C”	E.C.3.4.21.4	0.605	0.51
3M7U	E.C.3.4.21.12	0.600	0.59
2ALA	E.C.3.4.21.90	0.606	0.56
3PV3-„C”	E.C.3.4.21.107	0.698	1.44
3CS0	E.C.3.4.21.107	0.609	0.78
3LGW	E.C.3.4.21.107	0.408	0.18
1M6D-„D”	E.C.3.4.22.41	0.471	0.38
3BOW-„C”	E.C.3.4.22.53	0.676	1.04
1YP1	E.C.3.4.24	0.512	0.44
3V96	E.C.3.4.24.22	0.453	0.35
1WGZ-„C”	E.C.3.4.24.66	0.647	0.92
2OHQ	E.C.3.4.23.46	0.606	0.77
2A8L-„D”	E.C.3.4.25	0.442	0.31
3UNF-„C”	E.C.3.4.25.1	0.540	0.33
3PL1	E.C.3.5.1	0.590	0.66
2YWD	E.C.3.5.1.2	0.354	0.17
1SZZ-„D”	E.C.3.5.1.88	0.427	0.28
3DLD	E.C.3.5.1.88	0.540	0.52
3W4P	E.C.3.5.2.6	0.582	0.60
2ZC7	E.C.3.5.2.6	0.524	0.5
1BUE	E.C.3.5.2.6	0.487	0.39
3LUB-„C”	E.C.3.5.2.10	0.609	0.74
2EWO-„C”	E.C.3.5.3.12	0.520	0.50
3GVF	E.C.3.6.1.1	0.529	0.51
1YE8	E.C.3.6.1.15	0.440	0.28

3BHD-„D”	E.C.3.6.1.28	0.666	1.17
1W9K	E.C.3.6.4.1	0.732	1.33
3LLM	E.C.3.6.4.13	0.534	0.52
1C4X	E.C.3.7.1.8	0.455	0.37
**E.C.4.**		**22.7%**	
3P85	E.C.4.	0.507	0.44
1J93	E.C.4.1.1.37	0.545	0.55
3DTV-„C”	E.C.4.1.1.76	0.598	0.65
1UB3-„C”	E.C.4.1.2.4	0.556	0.57
2YW3-„C”	E.C.4.1.2.14	0.488	0.44
3C6X	E.C.4.1.2.47	0.428	0.31
3JYG-„C”	E.C.4.1.2.50	0.558	0.47
3H07-„D”	E.C.4.1.99.12	0.494	0.40
4DF0-„D”	E.C.4.2	0.466	0.35
1XKY	E.C.4.2.1.52	0.516	0.46
3EB2-„C”	E.C.4.2.1.52	0.553	0.58
2DW6-„C”	E.C.4.2.1.81	0.627	0.84
2PP3-„C”	E.C.4.2.1.156	0.623	0.68
1N7R	E.C.4.2.2.1	0.661	1.02
1X1L	E.C.4.2.2.12	0.792	1.63
2O11	E.C.4.2.3.5	0.672	0.85
1UM0-„C”	E.C.4.2.3.5	0.557	0.51
2DJ6-„C”	E.C.4.2.3.13	0.419	0.23
4LA5	E.C.4.2.3.118	0.645	0.79
3DAQ-„C”	E.C.4.3.3.7	0.506	0.48
1IV1-„D”	E.C.4.6.1.12	0.517	0.45
**E.C.5.**		**28.5%**	
3EEZ	E.C.5.	0.674	0.98
3C5Y-„C”	E.C.5.	0.455	0.33
1B9L-„C”	E.C.5.1.99.7	0.514	0.34
1VDN	E.C.5.2.1.8	0.488	0.43
3CGM	E.C.5.2.1.8	0.620	0.71
4G1K-„C”	E.C.5.3.1.1	0.539	0.48
1A0E-„D”	E.C.5.3.1.5	0.633	0.72
3LJK	E.C.5.3.1.9	0.623	0.65
1NSJ	E.C.5.3.1.24	0.399	0.28
3O22	E.C.5.3.99.2	0.414	0.24
3HWO-„D”	E.C.5.4.4.2	0.590	0.64
3GSE	E.C.5.4.4.2	0.681	0.90
1YBZ	E.C.5.4.99.5	0.549	0.47
**E.C.6.**		**14.2%**	
3IVV	E.C.6.	0.454	0.31
3TIG	E.C.6.	0.625	0.79
2RHS	E.C.6.1.1.20	0.741	1.44
1RY2	E.C.6.2.1.1	0.576	0.65

1V25	E.C.6.2.1.3	0.578	0.58
2NU9-„C”	E.C.6.2.1.5	0.643	0.86
1IOW	E.C.6.3.2.4	0.636	0.84
**E.C.7.**		**0%**	
4HUQ	E.C.7.	0.608	0.63
1JJ7	E.C.7.4.2	0.642	0.81
3IQY	E.C.7.4.2.8	0.742	1.58
2IPC	E.C.7.4.2.8	0.715	1.26
2PCJ	E.C.7.6.2	0.536	0.53

Tab. S1. Set of enzymes characterised by RD and K values. The symbols: “D” – enzyme in form of dimer, „C” – enzyme available in PDB in form of complex (oligomers). Values expressed in % - percentage of examples characterised by RD < 0.5.
